# Synthesis of Poly(olefin sulfone)s That Release Low-Molecular-Weight Bases by Light Absorption and Investigation of Their Photoinduced Depolymerization

**DOI:** 10.1021/acsomega.5c02541

**Published:** 2025-06-19

**Authors:** Sumie Takemura, Atsushi Seki, Khoa Van Le, Yumiko Naka, Takeo Sasaki

**Affiliations:** Department of Chemistry, Faculty of Science, Tokyo University of Science, 1-3 Kagurazaka, Shinjuku-ku, Tokyo 162-8601, Japan

## Abstract

Poly­(olefin sulfone)­s react with bases, leading to a depolymerization reaction, and convert themselves into monomers. A poly­(olefin sulfone) with an incorporated photobase generator undergoes depolymerization when exposed to light and subsequently heated. In previous studies, low-molecular-weight photobase generators have been mixed with poly­(olefin sulfone)­s or have been introduced onto their side chains. A photobase generator in a side chain decomposes when it is exposed to light, and a base attached to the main chain is generated. In the present study, we synthesized novel poly­(olefin sulfone)­s in which a photobase-generating moiety that releases a low-molecular-weight base is introduced into the side chains. In the polymers synthesized in this study, the photobase generator is uniformly dispersed throughout the polymer, and the generated bases can move freely. The photodegradation behavior of this polymer was investigated in detail.

## Graphical abstract

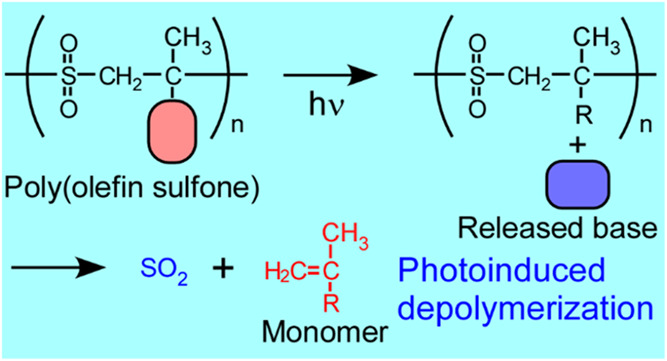

## Introduction

Photodegradable polymers have been developed for use in photolithography for some time.^1–8^ Research on their application in dismantlable adhesives^9,10^ and self-immolating protective coatings^11^ that decompose and vaporize when exposed to light has recently been reported. In addition to the polymer itself absorbing light and causing the main chain to break,^12,13^ some photodegradable polymer systems use photoacid or photobase generators to break down the polymer.^14–16^ The development of polymers that exhibit depolymerization or decomposition reactions in response to external stimuli is an active area of research. Among these, new polymers, such as those with end-caps introduced into polymers with low ceiling temperatures^7,8^ or those with cyclic structures,^2^ have been continuously reported. Depolymerization is defined as the process by which polymers revert to monomers through unzipping reactions, a process distinct from simple decomposition. Furthermore, polymers that generate low-molecular-weight compounds other than monomers through unzipping reactions are designated as self-immolative polymers.^2,6^ Polymers of this nature, which undergo decomposition at low temperatures in response to external stimuli, have recently garnered attention from the perspective of the sustainable development goals (SDGs). These polymers are the focus of extensive research, and their content has been thoroughly reviewed.^1–8^ A variety of depolymerizable and self-immolative polymers, including phthalaldehyde, polybenzcarbamate, and polydisulfide, have been investigated,^6–8^ and their development persists to the present day. Poly­(olefin sulfone)­s are polymers that depolymerize upon reaction with a base; thus, a mixture of a poly­(olefin sulfone) and a photobase generator exhibits photoinduced depolymerization.^17^ Poly­(olefin sulfone)­s are copolymers of an olefin monomer and sulfur dioxide (SO_2_).^18^ They are prepared by radical polymerization of olefin monomers in liquid SO_2_ (Figure 1). The olefins form a 1:1 charge-transfer complex with SO_2_, and an alternating copolymer of olefin and SO_2_ is thus obtained when they undergo radical polymerization.^19^ The sulfonyl group is electron-withdrawing; the hydrogen atoms of the hydrocarbons adjacent to the sulfonyl group therefore exhibit a high acidity. Consequently, these hydrogen atoms are abstracted by bases such as amines, which causes a depolymerization reaction in a poly­(olefin sulfone), leading to decomposition of the polymer into an olefin monomer and SO_2_ (Figure 1).^20^ We have previously incorporated a photobase generator into a poly­(olefin sulfone) and have studied a poly­(olefin sulfone) that depolymerizes when exposed to light.^19^ The corresponding films undergo a depolymerization reaction in the exposed area. In the case of poly­(olefin sulfone)­s prepared using olefin monomers with low boiling points, the film regions where depolymerization has occurred evaporate and are removed.^17^ In such photosensitive poly­(olefin sulfone) films, light irradiation leads to the generation of bases, which form latent images. Heating the film to ∼100 °C induces a hydrogen-withdrawal reaction, depolymerizing the polymer. Because the exposure operation and depolymerization can be carried out separately, there is less risk of contamination of the exposure device.

**Figure 1:**
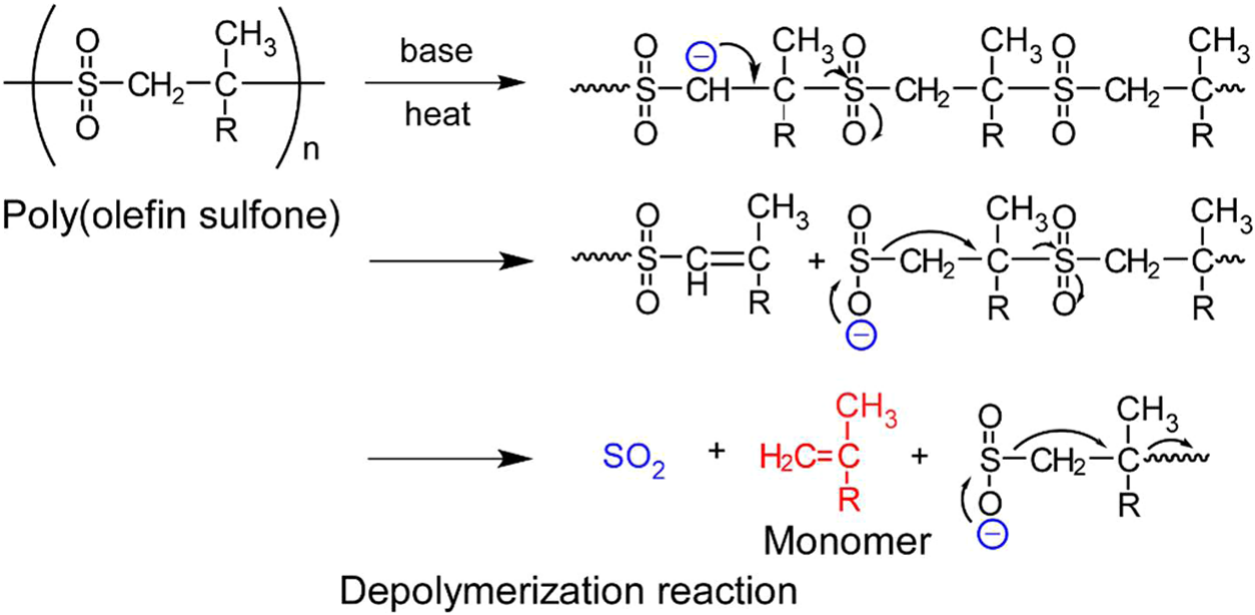
Depolymerization reaction of poly­(olefin sulfone)­s induced by the proton abstraction reaction by a base.

There are several ways to introduce photobase generators into poly­(olefin sulfone)­s, as shown in Figure 2: (a) A base can be generated by absorbing light and can be subsequently bound to the main chain;^15,21^ (b) a low-molecular-weight photobase generator can be mixed with the polymer;^9,17^ or (c) a free base can be released from the polymer main chain as a result of light absorption. Depolymerization occurs when the low-molecular-weight photobase generator is incorporated into a poly­(olefin sulfone) or when the olefin monomer with a photobase-generating site is polymerized. However, for a higher extent of photodegradation, it is desirable to have the photobase generator incorporated into the polymer’s molecular structure. In previous research, we synthesized poly­(olefin sulfone)­s in which bases are generated by light absorption and are bound to the polymer main chain.^15,21^ This process led to a greater extent of photodegradation.

**Figure 2:**
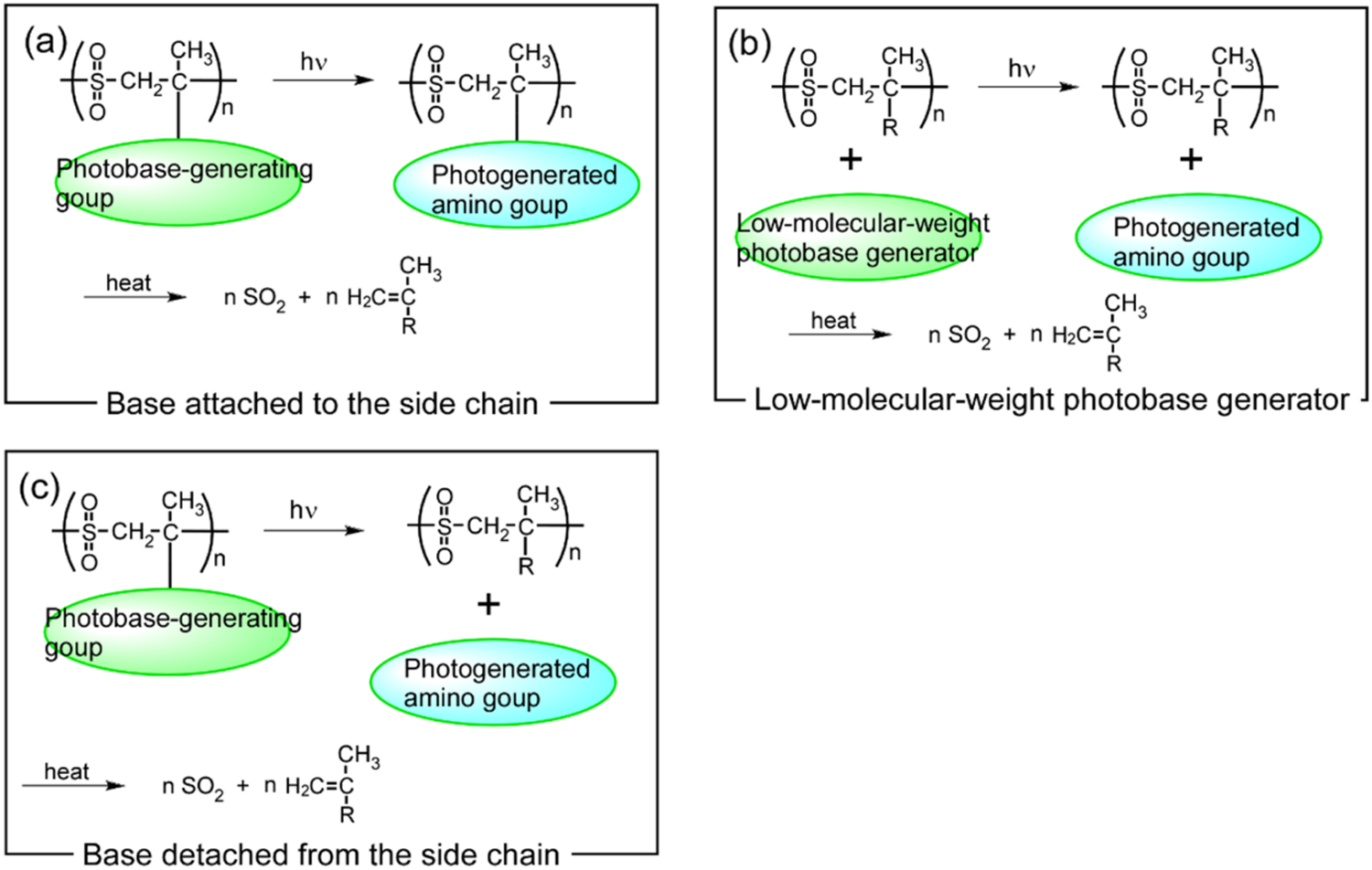
Introduction of photobase generators into poly­(olefin sulfone)­s.

We have also reported using base amplifiers to increase degradation efficiency.^16^ We speculate that if bases generated by light irradiation can separate from the polymer main chain and diffuse freely, then the efficiency of photodegradation can be further increased. Although the diffusion of the base is disadvantageous when high resolution is required, it is advantageous when the polymer film needs to be depolymerized all at once, such as in the case of a degradable adhesive. In this study, we synthesized poly­(olefin sulfone)­s with photobase generators that release free bases by absorbing light and examined their photodegradability.

## Methods

### Samples

In the present study, we synthesized poly­(olefin sulfone)­s by copolymerizing SO_2_ with olefin monomers bearing photobase generators. We chose polymer structures with methyl substituents in their main chain because such substituents limit the number of hydrogen atoms that can be extracted by a base, which increases the depolymerization rate.^17^ The structures of the poly­(olefin sulfone)­s used in this study are shown in Figure 3.

**Figure 3:**
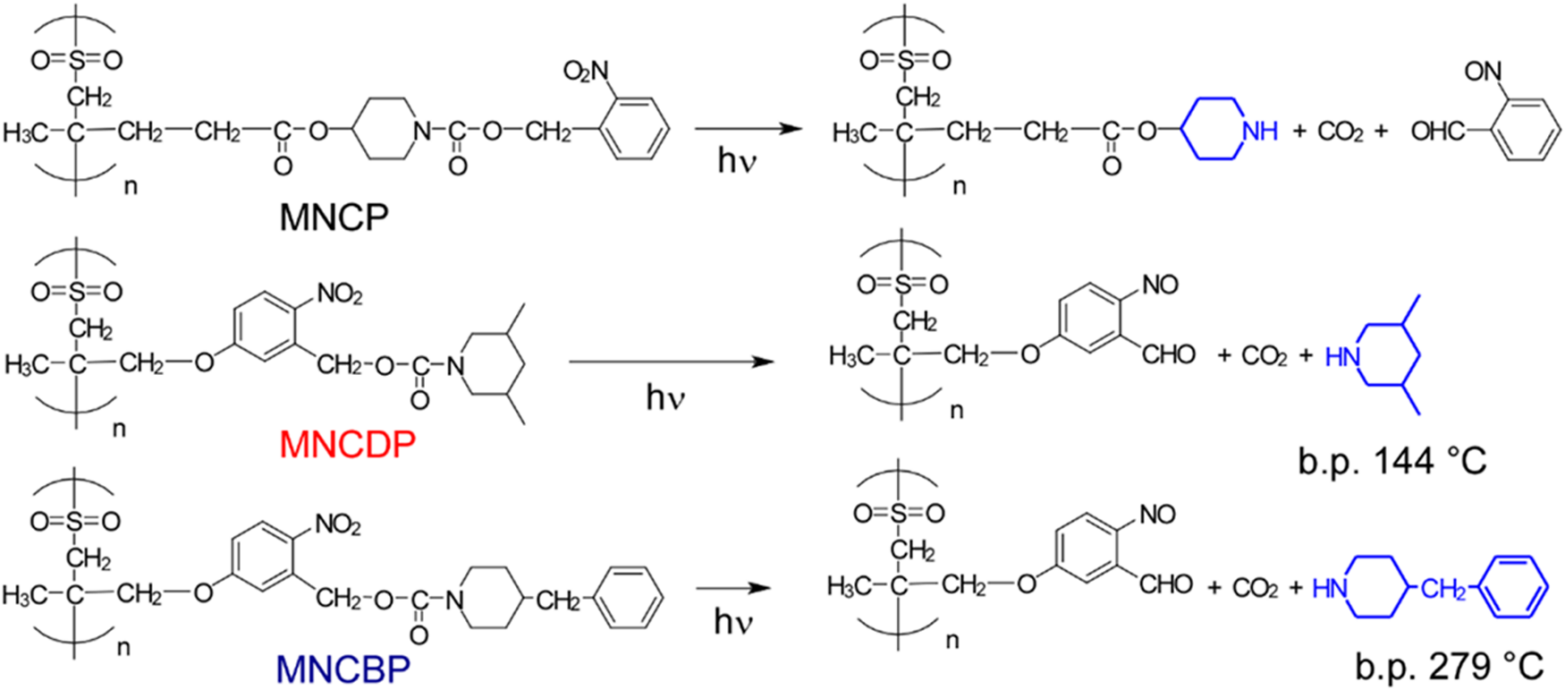
Structures of the poly­(olefin sulfone)­s synthesized in the present study.

*o*-Nitrobenzyl derivatives were chosen as photobase-generating units because *o*-nitrobenzyl derivatives are easy to synthesize and undergo base generation reactions with high efficiency.^4,22^ MNCP (poly­(2-nitrobenzyl 4-((4-methylpent-4-enoyl)­oxy)­piperidine-1-carboxylate sulfone)) is a previously reported polymer,^15^ and the base generated by light irradiation of MNCP remains bound to the polymer main chain. MNCDP (poly­(5-((2-methylallyl)­oxy)-2-nitrobenzyl 3,5-dimethylpiperidine-1-carboxylate sulfone)) and MNCBP (poly­(5-((2-methylallyl)­oxy)-2-nitrobenzyl 4-benzylpiperidine-1-carboxylate sulfone)) absorb light, releasing low-molecular-weight bases. When the MNCDP group absorbs light, it releases 3,5-dimethylpiperidine. Piperidine, a secondary amine with a p*K*_a_ of 11.12, is highly reactive in extracting hydrogen from poly­(olefin sulfone)­s.^15^ Introducing two methyl groups into piperidine increases the boiling point, making it less volatile. In addition, the MNCBP group releases 4-benzylpiperidine when it absorbs light. Because it has a phenyl group, benzylpiperidine has a high boiling point. The synthetic routes for the monomers are shown in Figure 4.

**Figure 4:**
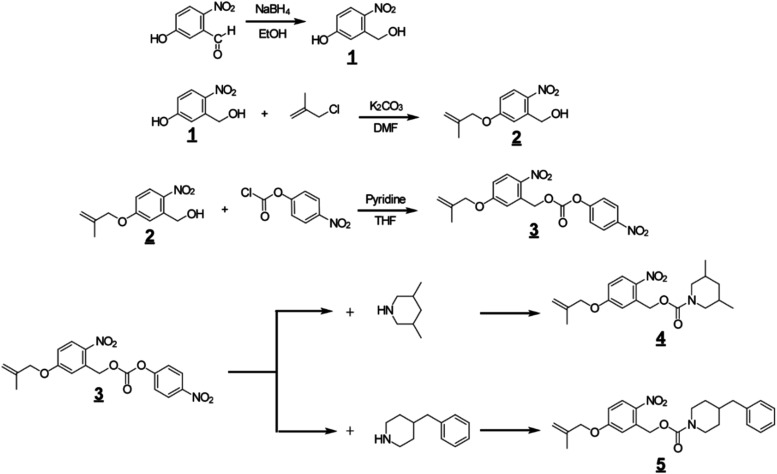
Synthetic route for the olefin monomers possessing base-release-type photobase generators.

#### Synthesis of 5-Hydroxymethyl-4-nitrophenol **(1)**

5-Hydroxy-2-nitrobenzaldehyde (16.0 g, 9.57 × 10^–2^ mol) and ethanol (200 mL) were dissolved in a 1000 mL three-necked round-bottom flask. With the temperature of this solution maintained at 0 °C, 8.36 g (2.20 × 10^–1^ mol) of NaBH_4_ dissolved in 100 mL of ethanol was added using an isobaric dropping funnel, and the resultant mixture was stirred for ∼30 min. The reaction was stopped by adding a HCl solution, and the ethanol was evaporated. The reaction product was dissolved in 500 mL of ethyl acetate, and the solution was washed with water. The ethyl acetate solution was then dehydrated with sodium sulfate. After the solvent was removed under reduced pressure, the white-yellowish crude crystals were recrystallized using a mixture of toluene, ethyl acetate, and hexane to obtain white crystals (6.39 g, yield 39.5%).

^1^ **H NMR (300 MHz, DMSO, ppm)**, δ8.05 (d, 1H, Ar-H), 7.18 (d, 1H, Ar-H), 6.74 (dd, 1H, Ar-H), 5.55 (br, 1H, Ar–OH), 4.82 (s, 2H, CH_2_OH)

#### [5-(2-Methyl-allyloxy)-2-nitro-phenyl]-methanol **(2)**

Potassium carbonate (8.77 g, 6.34 × 10^–2^ mol) and 5-hydroxymethyl-4-nitrophenol (4.98 g, 3.58 × 10^–2^ mol) were ground in a mortar and then dissolved in 200 mL of *N*,*N*-dimethylformamide in a 1000 mL three-necked flask. To this solution was added 2.68 g (2.96 × 10^–2^ mol) of 3-chloro-2-methyl-1-propene; the resultant mixture was then heated and stirred at 50 °C. After the reaction was completed, potassium carbonate was removed by filtration. A yellow liquid was obtained, to which aqueous HCl was added until the pH reached 2–3. The solution turned orange. The solvent was removed by evaporation. The crude product was purified using a silica-gel column with a mixed solvent of ethyl acetate and hexane (4:1) as an eluent, and a brown solid was obtained (4.90 g, yield 74.2%).

^**1**^ **H NMR (300 MHz, CDCl**_**3**_, **ppm)**, δ 8.24 (d, 1H, Ar-H), 7.24 (d, 1H, Ar-H), 6.94 (dd, 1H, Ar-H), 5.10 (s, 1H, = CH_2_), 5.00 (s, 1H, = CH_2_), 4.99 (d, 2H, CH_2_–OH), 4.56 (s, 2H, O–CH_2_-C­(CH_3_)=CH_2_), 2.63 (t, 1H, CH_2_–OH), 1.85 (s, 3H, CH_3_)

#### Carbonic Acid 5-(2-Methyl-allyloxy)-2-nitro-benzyl Ester 4-Nitro-phenyl Ester **(3)**

In a 300 mL three-necked flask, 4.80 g (2.15 × 10^–2^ mol) of [5-(2-methyl-allyloxy)-2-nitro-phenyl]-methanol (2) and 1.70 g (2.15 × 10^–2^ mol) of pyridine were dissolved in a small amount of tetrahydrofuran (THF); the resultant mixture was stirred while being cooled in an ice bath. 4-Nitrophenyl chloroformate (4.77 g, 2.36 × 10^–2^ mol) dissolved in THF was added to this solution using an airtight dropping funnel. After the addition was completed, the solution was heated and refluxed for 5 h. The results of silica-gel thin-layer chromatography using a mixture of ethyl acetate and chloroform (1:9) confirmed that all of the starting material was consumed. The reaction solution was washed with water, and the chloroform solution was dehydrated with sodium sulfate. The chloroform was evaporated, and a white powder was obtained by recrystallization using a mixture of ethanol and hexane (4.81 g, yield 57.6%).

^**1**^ **H NMR (300 MHz, CDCl**_**3**_, **ppm)**, δ 8.32 (dd, 2H, Ar-H), 8.25 (d, 1H, Ar-H), 7.45 (dd, 2H, Ar-H), 7.21 (s, 1H, Ar-H), 6.99 (d, 1H, Ar-H), 5.75 (s, 2H, Ar–CH_2_-O−), 5.12 (s, 1H, = CH_2_), 5.06 (s, 1H, = CH_2_), 4.58 (s, 2H, O–CH_2_-C­(CH_3_)=CH_2_), 1.86 (s, 3H, CH_3_)

#### *N*-[5-(2-Methyl-allyloxy)-2-nitrobenzyloxycarbonyl]-3,5-dimethylpiperidine (MNCDP Monomer, 4)

In a 500 mL three-necked flask, carbonic acid 5-(2-methyl-allyloxy)-2-nitro-benzyl ester 4-nitro-phenyl ester (3, 2.70 g, 6.95 × 10^–3^ mol), 3,5-dimethylpiperidine (0.810 g, 7.16 × 10^–3^ mol), 1-hydroxy benzotriazole (HOBt, 0.940 g, 6.96 × 10^–3^ mol), and 100 mL of THF were combined and refluxed for 8 h while stirring. After the reaction solution had cooled, chloroform was added, and the organic fraction was washed with a saturated sodium bicarbonate solution. The chloroform solution was dehydrated with sodium sulfate, and the solvent was evaporated. The product was purified by silica-gel column chromatography using a mixture of ethyl acetate and chloroform (1:9) as the eluent. The solvent was evaporated, and pale-yellow crystals were obtained (2.28 g, yield 90.8%).

^**1**^ **H NMR (300 MHz, CDCl**_**3**_, **ppm)** δ7.97 (br, 1H, Ar-H), 6.99 (br, 1H, Ar-H), 6.77 (br, 1H, Ar-H), 5.41 (br, 2H, Ar–CH_2_-O−), 4.64 (br, 2H, O–CH_2_-C­(CH3)=CH_2_), 3.97 (br, 2H, N–CH_2_), 2.15 (br, 2H, N–CH_2_), 1.80–0.634 (br, 8H, CH_2_, CH), 0.814 (br, 9H, C­(CH_3_))

**IR (KBr ν cm** ^**‑1**^ **)** 1701 (s, C = O str), 1523 (s, asymm N–O str), 1250 (s, asymm C–O str), 1227 (s, asymm C–O str), 1317 (s, SO_2_), 1141 (s, SO_2_)

The ^1^H NMR spectrum of the MNCDP monomer is shown in Figure S1.

#### *N*-[5-(2-Methyl-allyloxy)-2-nitrobenzyloxycarbonyl]-4-benzyl-piperidine (MNCBP Monomer, 5)

In a 300 mL three-necked flask, carbonic acid 5-(2-methyl-allyloxy)-2-nitro-benzyl ester 4-nitro-phenyl ester (**3**, 1.50 g, 3.86 × 10^–3^ mol), 4-benzyl -piperidine (0.68 g, 3.88 × 10^–3^ mol), HOBt (0.52 g, 3.85 × 10^–3^ mol), and 100 mL of THF were added and the resultant mixture was refluxed at 90 °C for 4 h. Chloroform was added to the reaction solution, which was washed with a saturated sodium bicarbonate solution. The chloroform solution was dehydrated with sodium sulfate. After the solvent was evaporated, the product was purified by silica-gel column chromatography with a mixture of ethyl acetate and chloroform (1:9) as the eluent. The solvent was evaporated, and a high-viscosity yellow oil was obtained (1.58 g, yield 96.3%).

^**1**^ **H NMR (300 MHz, CDCl**_**3**_, **ppm)** δ7.92 (br, 1H, Ar-H), 7.21–6.77 (m,s 5H, Ar-H), 5.38 (br, 2H, Ar–CH_2_-O−), 4.59 (br, 2H, O–CH_2_-C­(CH_3_)=CH_2_), 4.00 (br, 2H, N–CH_2_), 2.97 (br, 2H, N–CH_2_), 2.64 (br, 2H, N–CH_2_-Ar), 1.76 (br, 3H, CH_2_, CH), 1.07 (br, 2H, N–CH_2_)

**IR (KBr ν cm** ^**‑1**^ **)** 1704 (s, C=O str), 1523 (s, asymm N–O str), 1314 (s, SO_2_), 1234 (s, symm C–O str), 1140 (s, SO_2_)

The ^1^H NMR spectrum of the MNCBP monomer is shown in Figure S2.

### Polymerization

Poly­(olefin sulfone)­s were obtained by the radical polymerization of olefin monomers in liquid SO_2_. The boiling point of liquid SO_2_ is −10.2 °C, making it easy to liquefy. Therefore, experiments can be conducted in a closed system by using relatively simple experimental equipment. Liquid SO_2_ can dissolve various organic compounds. The polymerizations of olefin monomers in liquid SO_2_ are reviewed in an article^18^ and in the references cited therein. The olefin monomers were dissolved in liquid SO_2_ and polymerized with *tert*-butylperoxide (tBuOOH) as an initiator. The *tert*-butylperoxide acted as a redox initiator with SO_2_, producing a *tert*-butyloxy radical. The initiator (1.56 × 10^–2^ mol), olefin monomer (1.0 g, (2.4–3.0) × 10^–3^ mol), and SO_2_ (10 g, 1.6 × 10^–1^ mol) were added to a pressure-resistant glass tube at −196 °C. When the tube temperature was raised to above −70 °C, the frozen solution fused and the polymerization reaction initiated. The tube was then maintained at −13 °C for 1 h. After polymerization, the polymer was purified by precipitation from methanol, washed several times with methanol, and dried under a vacuum at room temperature. The presence of SO_2_ in the polymer was confirmed by Fourier transform infrared (FT-IR) spectroscopy (1311 and 1130 cm^–1^) and ^1^H nuclear magnetic resonance (^1^H NMR) spectroscopy. The ^1^H NMR spectra of the MNCDP, MNCBP, and MNCP polymers are shown in Figures S3–S5, respectively. The molecular weights and thermal properties of the obtained polymers are listed in Table 1. The GPC curves, DSC curves, and TGA curves of the MNCDP, MNCBP, and MNCP polymers are shown in Figures S6–S8, respectively.

**Table 1:** Thermal Properties of the Polymers Used in the Present Study

poly(olefin sulfone)	*M_n_*	*M*_w_	*M*_w_/*M_n_*	*T*_g_ (°C)^a^	*T*_d_ (°C) (10 wt % loss)
MNCP	41,000	140,000	3.4	84	160
MNCDP	187,000	587,000	3.1	83	191
MNCBP	157,000	343,000	2.2	104	190

a The glass-transition temperatures were determined from the first scan data of the DSC measurement because the poly­(olefin sulfone)­s thermally degraded during the measurement.

### Measurement

Infrared spectra were obtained with a Jeol JIR-5500 FT-IR spectrophotometer. The number-average molecular weights (*M_n_*) of the polymers were determined by gel-permeation chromatography (GPC; Tosoh HLC-8220 with a Super Multipore HZ-M column, THF eluent), and the glass-transition temperatures (*T*_g_) were determined by differential scanning calorimetry (DSC; Mettler, DSC822e). The decomposition temperature was confirmed by thermogravimetric analysis (TGA; TA Instruments, Hi-Res TGA2950). ^1^H NMR spectra were acquired by using a Jeol EX-400 spectrometer (500 MHz). The polymer films were irradiated using a 250 W superhigh-pressure mercury lamp (Ushio, SX-UI250HQ). The intensity of the ultraviolet (UV) light was maintained at 32 mW/cm^2^ (at 365 nm) and 23 mW/cm^2^ (at 254 nm). A color filter (Toshiba, UVD-36C) and an interference filter were used to obtain monochromatic light. The thicknesses of the films were measured by atomic force microscopy (AFM; Keyence, VN-8000).

## Results and Discussion

### Photochemical Reactions of the Base-Release-Type Photobase-Generating Groups

The UV–visible (UV–vis) absorption spectra of chloroform solutions of the MNCDP and MNCBP monomers synthesized in the present study are shown in Figure 5.

**Figure 5:**
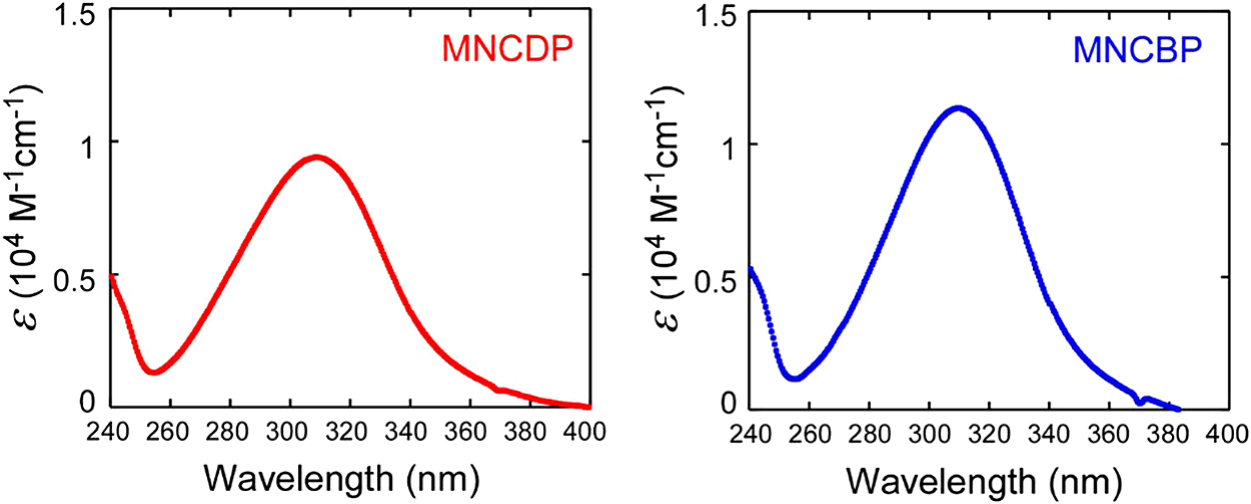
Molar absorption coefficients (ε) of the olefin monomers MNCDP and MNCBP in chloroform.

The spectra of both monomers show absorptions at 254 and 366 nm (superhigh-pressure mercury lamp). The photochemical reactions of the photobase generators in polymer films were investigated via IR absorption measurements. The polymers (2 mg) were dissolved in chloroform (100 μL), and the resultant solutions were dropped onto KBr plates. The KBr plates were heated at 80 °C for 1 min, evaporating the chloroform. UV light (254–366 nm) from the superhigh-pressure mercury lamp passed through a band-pass filter (UTVAF-50S-33U, Sigmakoki, Japan) and irradiated the films, and the changes in the films’ IR spectra were investigated (Figure 6).

**Figure 6:**
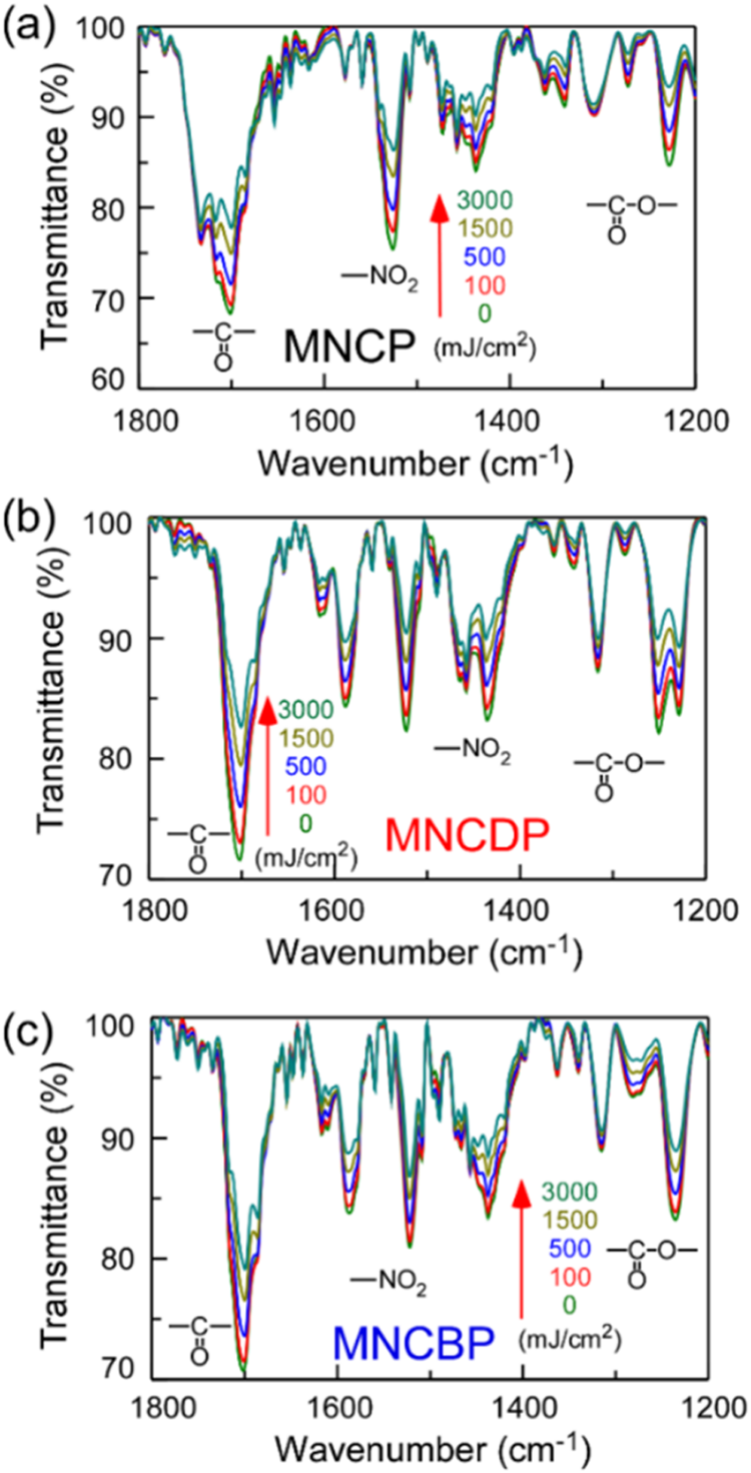
IR absorption spectra of (a) MNCP, (b) MNCDP, and (c) MNCBP films coated onto KBr plates after irradiation with UV light. Wider-range spectra are shown in Figure S9.

The absorbance of the carbonyl (−CO–, 1701 cm^–1^), ester (−CO–O–, 1230 cm^–1^), and nitro (–NO_2_, 1523 cm^–1^) groups decreased with increasing irradiation time, indicating that the photobase-generating groups undergo photodegradation reactions (Figure 3). This photochemical reaction removes the carbonyl groups as a CO_2_ gas and converts the nitro groups to nitroso (–NO) groups.

### Photoinduced Depolymerization of the Poly­(olefin sulfone)­s Possessing Photobase-Generating Groups

Polymer films were prepared by dropping a chloroform solution of MNCP onto KBr plates; the films’ IR spectra were recorded after the films were maintained at a temperature between 80 and 100 °C for 30 min (Figure 7(a)).

**Figure 7:**
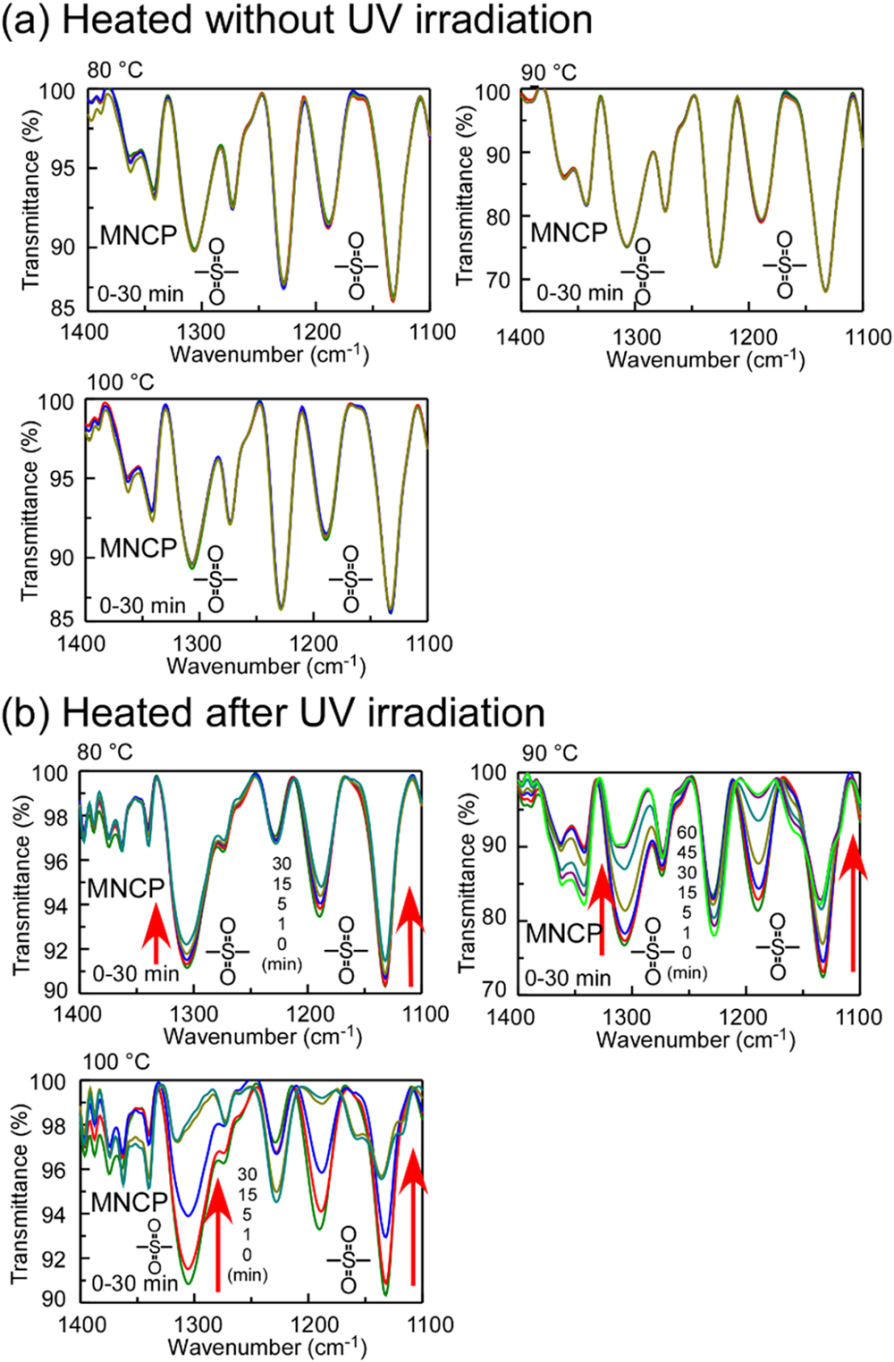
IR absorption spectra of MNCP polymer films coated onto KBr plates: (a) Films heated for 30 min at 80, 90, and 100 °C without UV irradiation; (b) films heated after UV irradiation at 3000 mJ/cm^2^ at 80, 90, and 100 °C for 30 min. Wider-range spectra are shown in Figure S10.

Poly­(olefin sulfone)­s have low thermal degradation temperatures because, when they are heated, their sulfonyl group withdraws hydrogen from the polymer main chain, causing depolymerization.^23^ However, we observed almost no change in the IR spectra during 30 min of heating at 80 or 100 °C, confirming that MNCP is not decomposed by simply heating at these temperatures. The MNCP film was irradiated with UV light (254–366 nm, 3000 mJ/cm^2^) from a superhigh-pressure mercury lamp with a color filter and then heated. The time-dependent changes in the IR absorption spectra of the films are shown in Figure 7(b). The intensity of the absorption peaks for the sulfonyl group (1311 and 1130 cm^–1^) decreased with an increasing heating time, confirming that the main chain of the poly­(olefin sulfone) was depolymerized. The residual ratios for the sulfonyl group, as obtained from the IR absorption spectra (1130 cm^–1^), are plotted as a function of the heating time in Figure 8.

**Figure 8:**
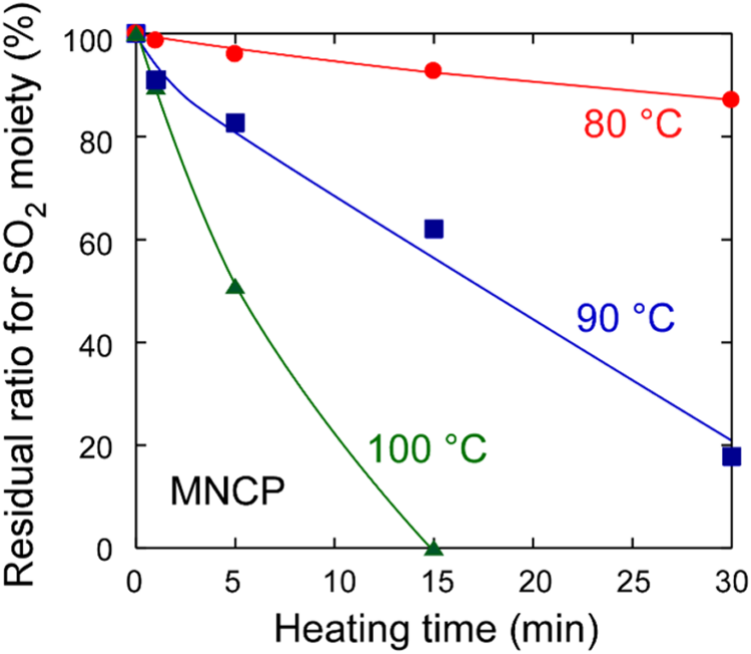
Residual ratio for sulfonyl moiety in MNCP polymer films as a function of heating time after UV irradiation at 3000 mJ/cm^2^. The heating temperatures were 80 °C (red), 90 °C (blue), and 100 °C (green). The IR absorption spectra used to calculate the residual ratio are shown in Figure S11.

The results in Figure 8 show that the amount of sulfonyl groups decreases almost linearly with an increasing heating time. The secondary amino group produced by photoirradiation induces depolymerization by removing hydrogen from the main chain of the poly­(olefin sulfone). Nonetheless, because the photogenerated amino group is bonded to the main chain, it is not lost by vaporization even when the sample is heated; the polymer is degraded in proportion to the heating time.

The same experiments were conducted for MNCDP, which releases free bases through light absorption. Figure 9(a) shows the change in the IR spectra when the MNCDP films were maintained at temperatures between 100 and 130 °C for 30 min. The absorption peaks of the sulfonyl group did not substantially change, confirming that the MNCDP polymer was not depolymerized simply by heating. The IR absorption spectra were measured after the MNCDP film was irradiated with UV light and heated. The IR absorption spectra are shown in Figure 9(b). The absorption peaks of the sulfonyl group decreased in intensity with an increasing heating time, confirming that the polymer was depolymerized. The ^1^H NMR spectra of the MNCDP polymer before and after it was subjected to UV irradiation and heating are shown in Figure S14. When the polymer film was irradiated with UV light and then heated, its ^1^H NMR spectrum changed substantially. The signals that appeared after light irradiation coincide with the signals in the ^1^H NMR spectrum of the monomer, indicating that a large portion of the polymer was depolymerized to monomers.

**Figure 9:**
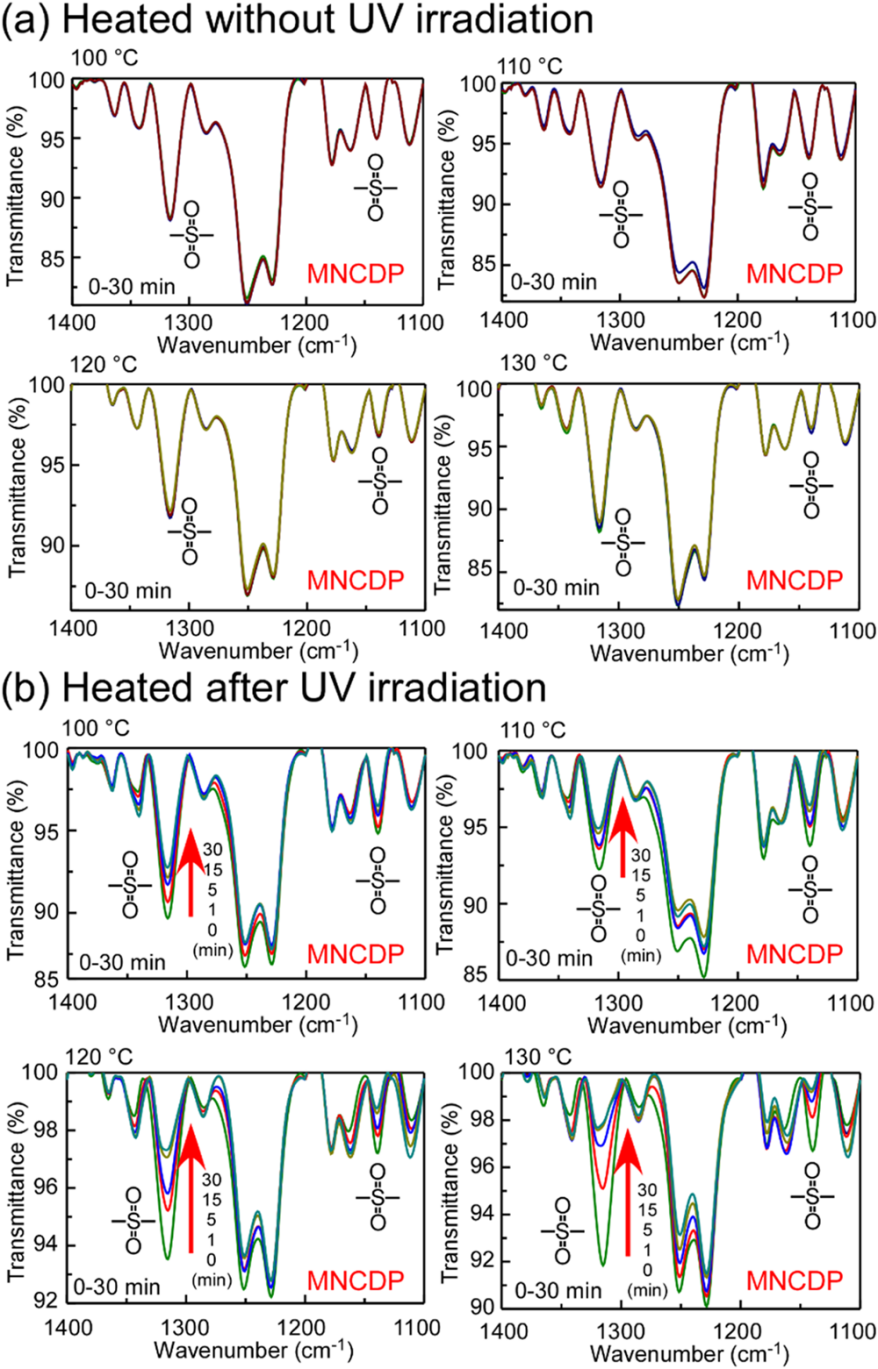
IR absorption spectra of MNCDP polymer films coated onto KBr plates: (a) Film heated for 30 min at 100, 110, 120, and 130 °C without UV irradiation; (b) film heated after UV irradiation of 3000 mJ/cm^2^ at 100, 110, 120, and 130 °C for 30 min. Wider-range spectra are shown in Figure S10.

The residual ratios for the sulfonyl groups, as obtained from the IR absorption spectra (1130 cm^–1^), are plotted as a function of the heating time in Figure 10. The ratios decreased nonlinearly with heating time, and the sulfonyl groups did not completely disappear after 30 min. This result is markedly different from that for the MNCP polymer, in which the base is bound to the main chain. In the MNCDP polymer, the photopolymerization reaction is considered to be suppressed because the base generated by the absorption of light is lost from the system by evaporation due to heating (the boiling point of 3,5-dimethylpiperidine is 144 °C).

**Figure 10:**
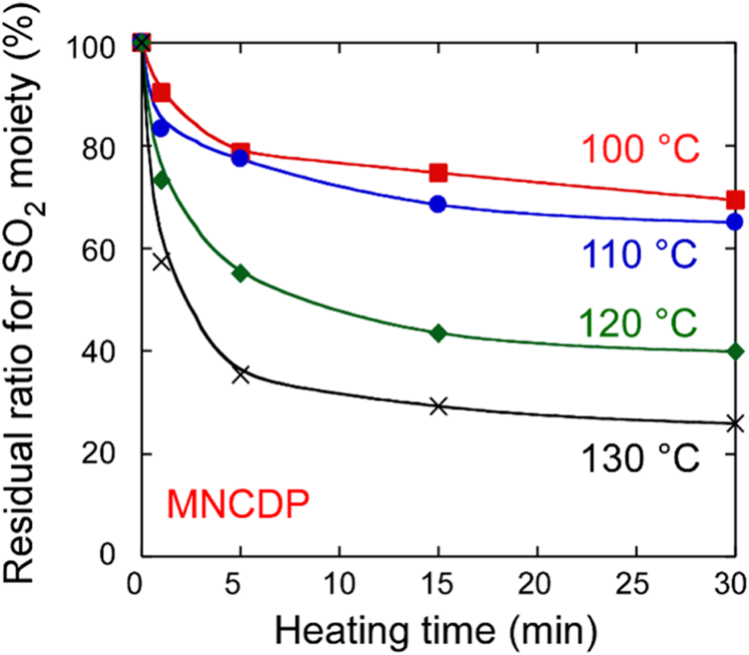
Residual ratio for the sulfonyl moiety in MNCDP polymer films as a function of heating time after UV irradiation at 3000 mJ/cm^2^. The heating temperatures were 100, 110, 120, and 130 °C. The IR absorption spectra used to calculate the residual ratio are shown in Figure S12.

The decomposition reaction was rapid for the first 5 min after heating began but then slowed; after 15 min, the decomposition reaction almost stopped. The low-molecular-weight base reacted rapidly with the poly­(olefin sulfone) main chain because the base could move freely. We speculate that the final depolymerization rate was low because the low-molecular-weight base evaporated due to heat and was lost from the system. Therefore, we designed a photobase generator that releases a base component with a high boiling point. MNCBP releases 4-benzylpiperidine (boiling point of 279 °C). Figure 11(a) shows the changes in the IR spectra when MNCBP films were maintained at 100–130 °C for 30 min.

**Figure 11:**
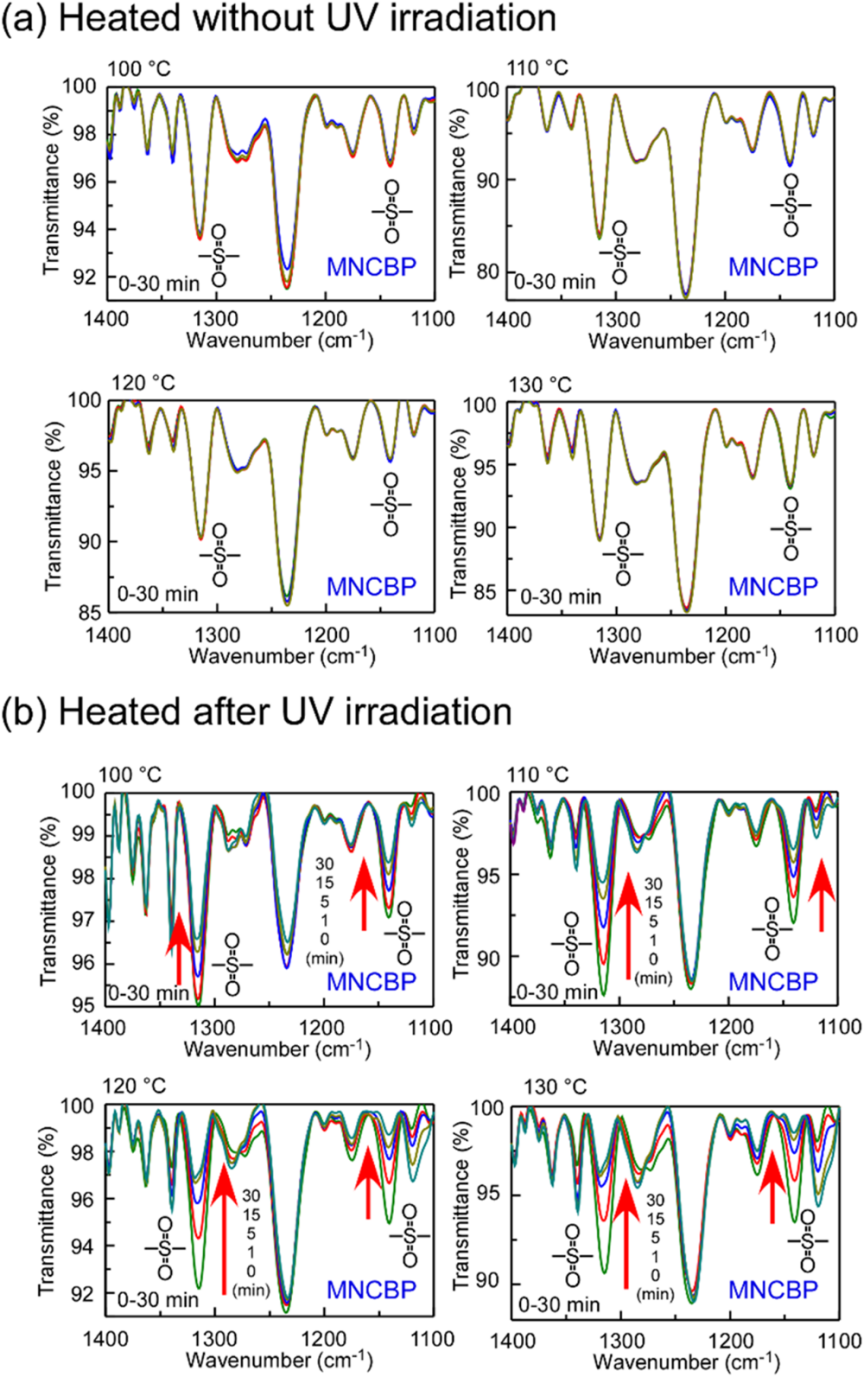
IR absorption spectra of MNCBP polymer films coated onto KBr plates: (a) Films heated for 30 min at 100, 110, 120, and 130 °C without UV irradiation; (b) films heated after UV irradiation at 3000 mJ/cm^2^ at 100, 110, 120, and 130 °C for 30 min. Wider-range spectra are shown in Figure S10.

The absorption peaks for the sulfonyl group showed almost no change, confirming that the MNCBP polymer did not decompose simply by heating. The MNCBP film was irradiated with UV light and heated. Its IR absorption spectra are shown in Figure 11(b). The intensity of the absorption peaks for the sulfonyl group decreased with an increasing heating time, confirming that the polymer was depolymerized. The ^1^H NMR spectra of MNCBP polymer films before and after UV irradiation and heating are shown in Figure S15. When an MNCBP polymer film was irradiated with UV light and then heated, its ^1^H NMR spectrum changed substantially. The signals that appeared after light irradiation coincide with the ^1^H NMR spectrum of the monomer, indicating that a large portion of the polymer was depolymerized to monomers. Figure 12 shows the residual ratios for the sulfonyl group, as obtained from the IR absorption spectra (1130 cm^–1^), plotted as a function of the heating time.

**Figure 12:**
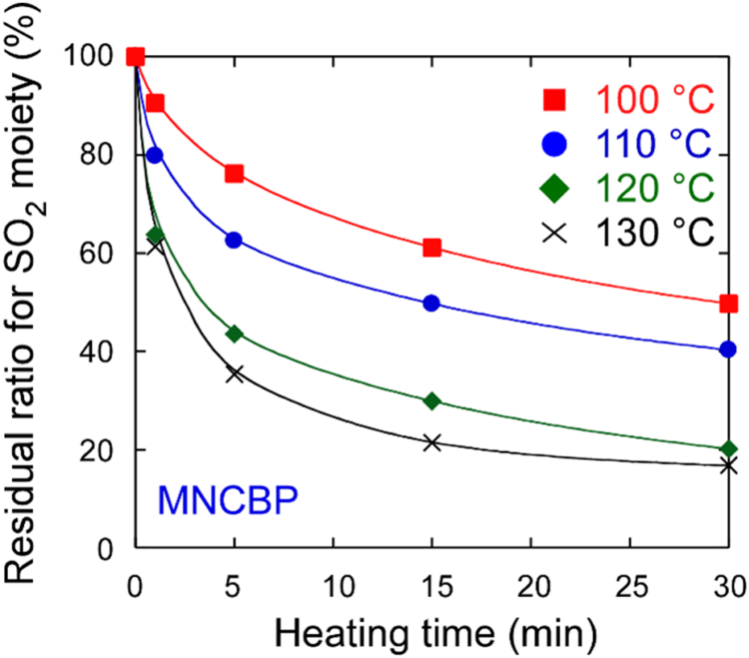
Residual ratio for the sulfonyl moiety in MNCBP polymer films as a function of the heating time after UV irradiation at 3000 mJ/cm^2^. The heating temperatures were 100, 110, 120, and 130 °C. The IR absorption spectra used to calculate the residual ratio are shown in Figure S13.

The residual ratios for sulfonyl groups decreased nonlinearly with increasing heating time, but the sulfonyl groups did not completely disappear after 30 min. The boiling point of 4-benzylpiperidine (279 °C) is higher than the heating temperature (130 °C). However, the concentration of the photogenerated 4-benzylpiperidine is low in the film, and it was considered that the amine evaporated from the polymer film and could not depolymerize the polymer completely. However, the final residual ratio for the sulfonyl groups was lower than that observed for the MNCDP polymer. There is a difference in the side-chain structure adjacent to the backbone between the MNCP, MNCDP, and MNCBP. However, the existence of the ethyl and the methyl carbons between the polymer main chain and the photobase-generating groups makes it possible for the piperidine to come close to the polymer main chain. The difference in the depolymerization behaviors is attributed to the difference in the mobilities of the photogenerated base. The residual ratios for the sulfonyl groups in the MNCDP and MNCBP polymers are compared in Figure 13.

**Figure 13:**
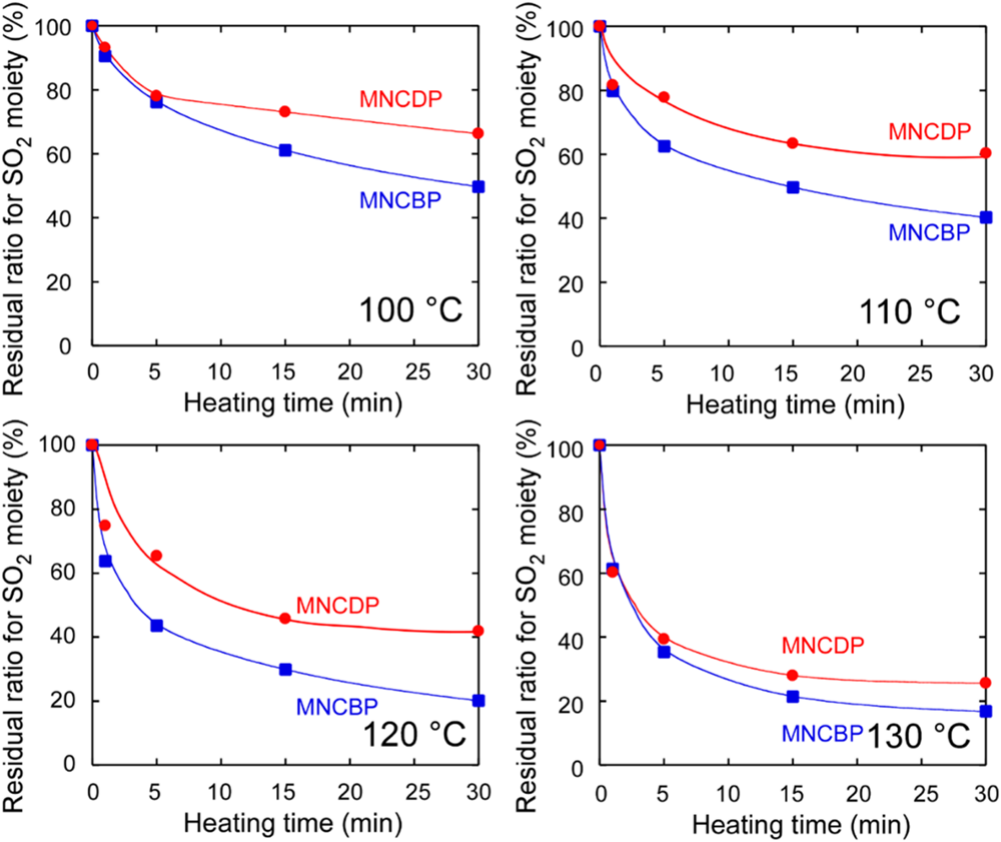
Comparison of the decomposition rates of MNCDP polymer and MNCBP polymer heated at 100, 110, 120, and 130 °C after UV irradiation at 3000 mJ/cm^2^.

The rate of decomposition of MNCBP was higher than that of MNCDP; however, the final residual ratio for MNCBP was ∼20%, indicating that it did not decompose completely. This lack of complete decomposition is attributed to the evaporation of the free base. However, the free base from MNCBP has a higher boiling point than that from MNCDP; therefore, the free base from MNCBP evaporated less quickly and remained in the system.

### Photoinduced Depolymerization of Polysulfone Copolymers Composed of a Volatile Monomer and Olefins Possessing Photobase-Generating Groups

The films of polymers that undergo photoinduced depolymerization can exhibit vaporization of the exposed area.^17,24,25^ These polymers are useful in dismantlable adhesives and self-immolating protective coatings. Poly­(olefin sulfone)­s composed of a volatile olefin monomer incorporated with the photobase generator MNCBP were synthesized, and their photoinduced decomposition was investigated. Ternary copolymers were synthesized from an MNCBP monomer, 2-methyl-1-pentene, and SO_2_ (Figure 14).

**Figure 14:**
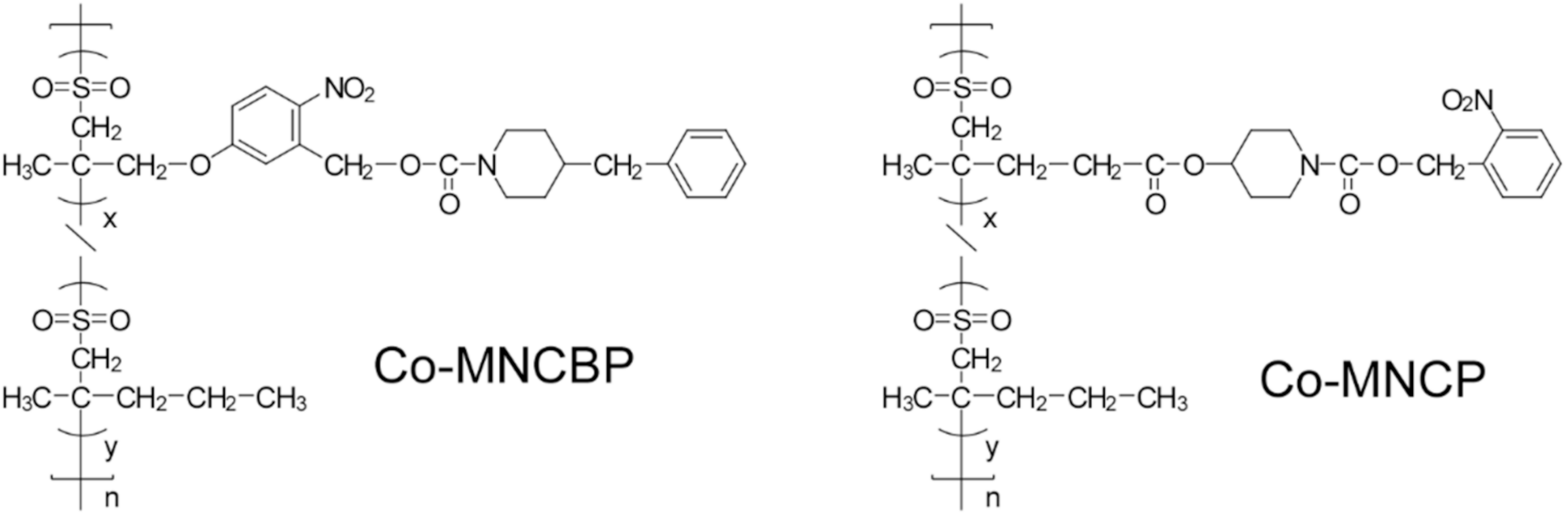
Structures of random copolymers of photobase-generating monomers, 2-methylpentene, and SO_2_. Co-MNCBPs with three different copolymerization ratios were synthesized; Co-MNCBP1 (*x*:*y* = 1:33), Co-MNCBP2 (*x*:*y* = 1:17), and Co-MNCBP3 (*x*:*y* = 1:8). The copolymerization ratio of Co-MNCP was 1:12.

2-Methylpentene has a low boiling point (61 °C); therefore, it volatilizes and is removed when it is depolymerized. Consequently, the region exposed to light is expected to be removed by volatilization when the copolymer of 2-methyl-1-pentene and a photobase-generating monomer are irradiated with light and heated. The thermal decomposition temperature (*T*_d_), glass-transition temperature (*T*_g_), molecular weight, and copolymerization ratio of the copolymers synthesized in the present study are shown in Table 2. ^1^H NMR spectra of the copolymers and the signals used for the calculation of the copolymerization ratios are shown in Figures S16–S21.

**Table 2:** Thermal Properties of the Copolymers Used in the Present Study

	*T*_d_ (°C) 10% wt loss	*T*_g_ (°C)	*M_n_*	*M*_w_	*M*_w_/*M_n_*	copolymerization ratio (*x*:*y*)
Co-MNCBP1	149	89.5	46,000	320,000	7.0	1:33
Co-MNCBP2	154	85.2	63,000	130,000	2.1	1:17
Co-MNCBP3	135	82.6	44,000	160,000	3.6	1:8
Co-MNCP	151	103	134,000	337,000	2.5	1:12

Two milligrams of each of these copolymers was dissolved in 10 mL of chloroform, and 2 mL of the resultant solution was dropped onto a KBr plate and heated to dryness, forming a film. This film was irradiated with UV light (254–366 nm, 3000 mJ/cm^2^) from a superhigh-pressure mercury lamp equipped with a color filter and then heated. The IR absorption spectra of the copolymers before and after UV-light irradiation and heating at 120 °C are shown in Figure 15.

**Figure 15:**
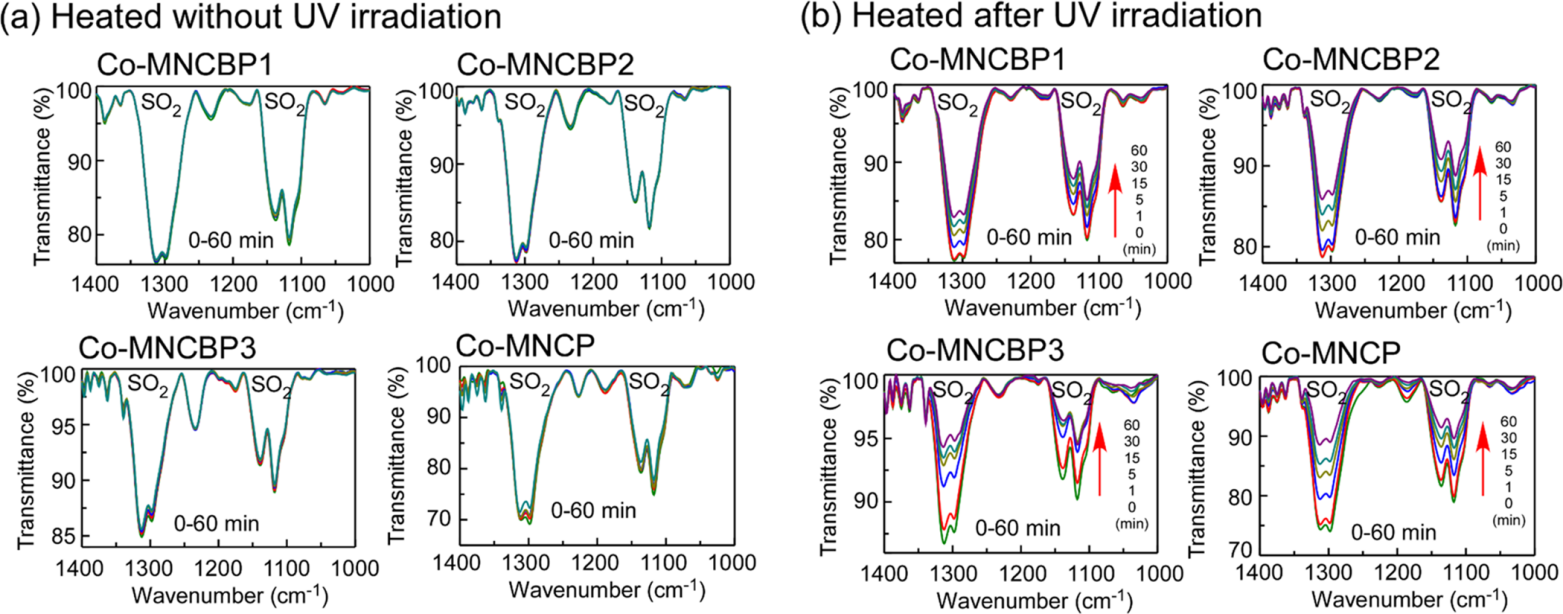
IR absorption spectra of copolymer films: (a) Films heated without irradiation at 120 °C for 60 min; (b) films heated after UV irradiation at 3000 mJ/cm^2^ at 120 °C for 60 min.

When the copolymer films were heated at 120 °C in the absence of UV light, absorption by their sulfonyl group showed almost no change even after 60 min of heating. However, when the copolymers were irradiated with UV light at 3000 mJ/cm^2^ and then heated at 120 °C, the absorption by the sulfonyl group decreased with increasing heating time. Figure 16 shows the results of the change in the residual ratio for the sulfonyl group (1139 cm^–1^), plotted as a function of the heating time.

**Figure 16:**
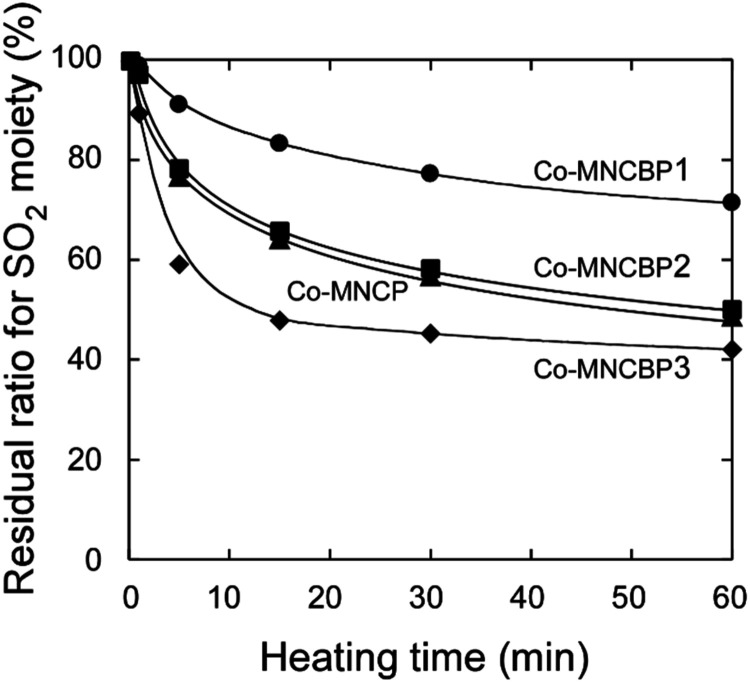
Residual ratio for the sulfonyl moiety in copolymer films as a function of the heating time after UV irradiation at 3000 mJ/cm^2^. The heating temperature was 120 °C.

The copolymers with the highest photobase generator incorporation rate (Co-MNCBP3) showed the fastest decrease in the number of sulfonyl groups and the largest final decrease in the number of sulfonyl groups. However, even Co-MNCBP3 showed a decrease of only ∼60%, which we speculatively attribute to an insufficient amount of base generated by light irradiation, enabling the base to evaporate from the system before it depolymerizes a large amount of the polymer. The thicknesses of the Co-MNCBP2 films were measured using AFM. Copolymer samples of 30 mg were dissolved in 1 mL of chloroform and spin-coated onto glass substrates (Figure 17(a)).

**Figure 17:**
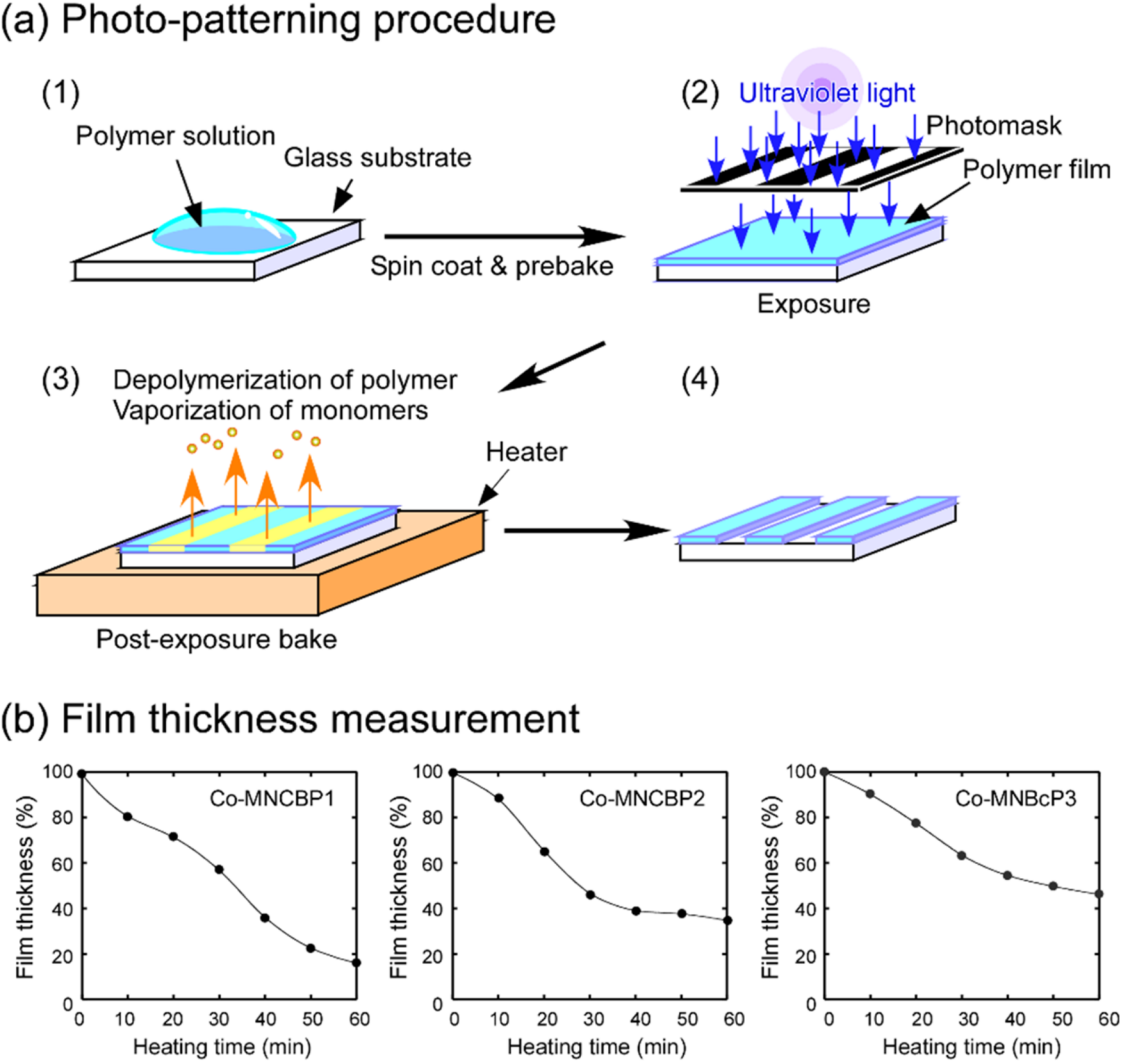
(a) Photopatterning experiments on copolymer films. (b) Residual ratios of the polymer films, as measured by AFM, plotted as a function of the heating time. UV light with a fluence of 10 J/cm^2^ was irradiated. The heating temperature was 120 °C.

The films were heated at 80 °C for 5 min to evaporate chloroform, and films with thicknesses of 300–500 nm were obtained. After the polymer films were irradiated with UV light at a fluence of 10 J/cm^2^, they were heated to 120 °C on a hot plate. The change in the film thicknesses is shown in Figure 17(b). The film thicknesses began to decrease immediately when the films were heated; after 60 min, they had decreased to 20–40% of their initial thicknesses. These results indicate that the poly­(olefin sulfone)­s were depolymerized by the bases generated by light irradiation and that SO_2_ and 2-methyl-1-pentene were volatilized. The final residual ratio for the film thickness in Co-MNCBP1 was lower than that in Co-MNCBP3. This lower final residual ratio was caused by the amount of vaporizable monomer (2-methylpentene) in the polymers. In Figure 16, the reduction in SO_2_ groups was the greatest for Co-MNCBP1, followed by those for Co-MNBP2 and Co-MNCBP3. However, in Figure 17, the decrease in film thickness was in the order of Co-MNBP1, Co-MNBP2, and Co-MNBP3, which is the reverse of that shown in Figure 16. This is due to the content of volatile components (2-methyl-1-pentene). Co-MNCBP1 contains the highest amount of volatile components, resulting in a larger change in film thickness upon decomposition. If the rate of depolymerization does not increase because of the volatilization of the base, then irradiating the glass-substrate side with light may suppress volatilization of the base and increase the rate of depolymerization. We therefore conducted an experiment in which light was irradiated from the glass-substrate side (Figures S22 and S23). However, we observed no difference between irradiating the film from the air side and irradiating it from the glass-substrate side. The films used in this experiment were thin; thus, the effect of the film thickness on the evaporation of the base was thought to be small.

Next, we patterned the Co-MNCBP2 polymer film by irradiating it with UV light through a photomask (Toppan Test Chart No. 1-N, Figure 18(a)) and then heating it (Figure 18(b)). The exposed areas were depolymerized by heating, and the monomers were removed by evaporation. AFM was used to conduct patterning experiments on Co-MNCBP2 and Co-MNCP films, which showed a similar decrease in sulfonyl groups after UV-light irradiation and heating (Figure 16). Co-MNCP and Co-MNCBP2 were spin-coated onto glass plates. After these films were irradiated with UV light in 30 μm-wide lines through a photomask, the exposed areas were heated to 120 °C, causing depolymerization and evaporation. The resultant grooves were then observed by AFM (Figure 18(c,d)).

**Figure 18:**
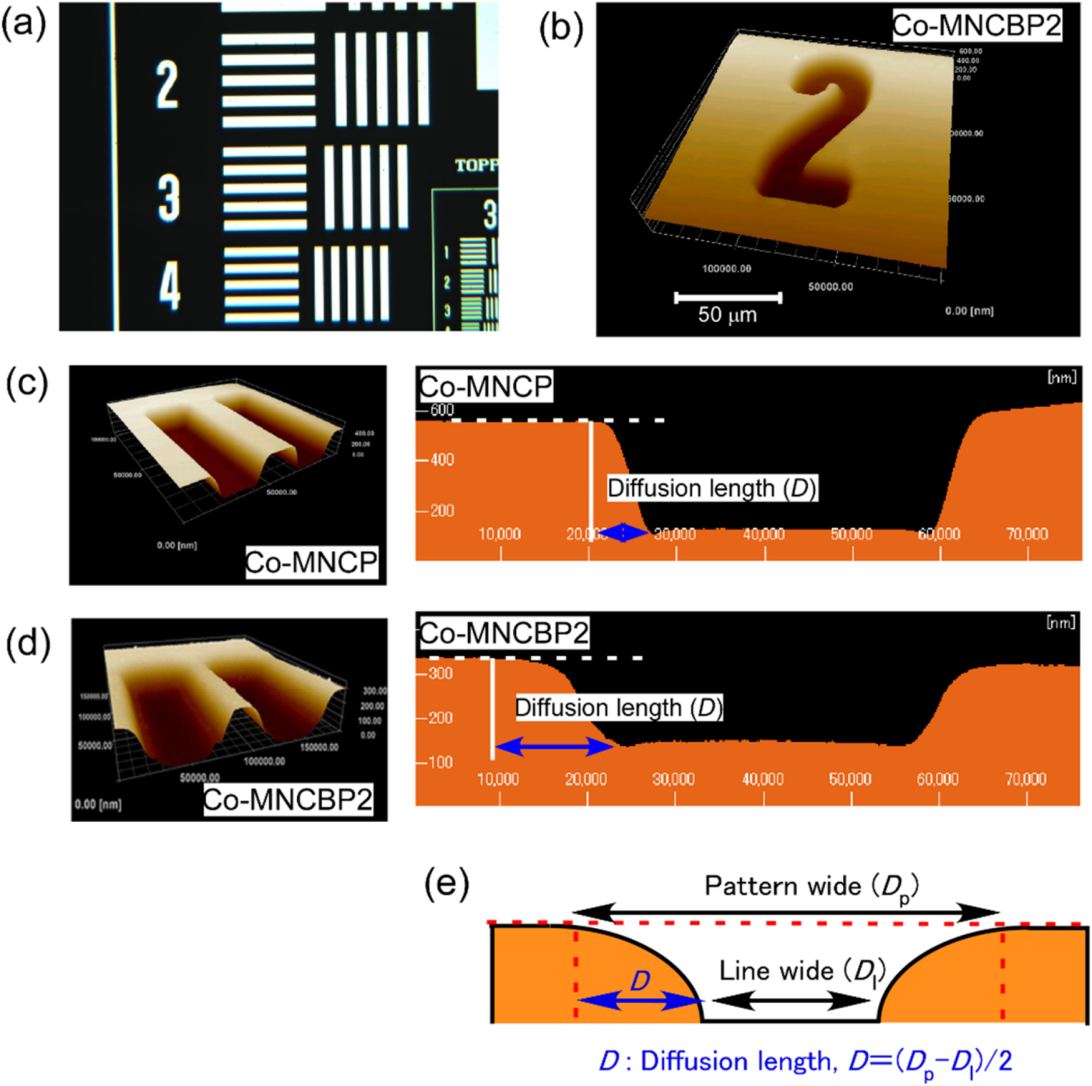
Photopatterning experiments on copolymer films. (a) Test pattern. (b) AFM image of a Co-MNCBP2 film after UV irradiation at 10 J/cm^2^ and heating at 110 °C for 60 min. (c) AFM image of a Co-MNCP film after UV irradiation at 10 J/cm^2^ and heating at 110 °C for 60 min. (d) AFM image of a Co-MNBP2 film after UV irradiation at 5000 mJ/cm^2^ and heating at 110 °C for 60 min. (e) Definition of the diffusion length.

Compared with the degraded lines on the Co-MNCP film, those on the Co-MNCBP2 film are wider. We attribute these wider lines to diffusion of the free base within the polymer film. If we assume that the difference between the width of the bottom and the width of the top of the formed depression is the diffusion distance *D* for the base (Figure 18(e)), then *D* = 6.8 μm for Co-MNCP and *D* = 14.4 μm for Co-MNCBP2. Although poly­(olefin sulfone)­s with free bases are not suitable for microfabrication, they are ideal for applications in which a large area needs to be decomposed by light irradiation such as photoremovable adhesives and photodegradable paints.

## Conclusions

We synthesized poly­(olefin sulfone)­s that release low-molecular-weight bases by light absorption and investigated their depolymerization by light irradiation and heating. The generated bases can move freely within the polymer films; thus, the rate of polymer decomposition at the beginning of the heating is high. However, the heating process causes the generated bases to evaporate, inhibiting decomposition of the polymer films. Evaporation can be suppressed by increasing the molecular weight of the free bases. The bases diffuse within the polymer films, causing degradation even in areas not exposed to light. As a result, the poly­(olefin sulfone)­s are not suitable for microfabrication but are ideal for applications, such as photoremovable adhesives and photodegradable paints.

## Supplementary Material



## Notes

1H NMR spectra of monomers and polymers of MNCDP, MNCBP, and MNCP; calculation procedure of decomposition rate of polymers; ^1^H NMR spectra of polymers before and after UV irradiation; ^1^H NMR spectra of copolymers Co-MNCBP 1, 2, and 3 and the NMR signals used for the calculation of the copolymerization ratios; changes in film thickness when UV light is irradiated from the air side of the film and from the glass-substrate side (PDF)
