# Supplementary material for: Synthesis of Poly(olefin sulfone)s That Release Low-Molecular-Weight Bases by Light Absorption and Investigation of Their Photoinduced Depolymerization
Source: ACS Omega. 2025 Jun 19;10(25):27238–49. doi: 10.1021/acsomega.5c02541 (PMC12223898; doi:10.1021/acsomega.5c02541)
Supplement: Supplementary file 1 [file ao5c02541_si_001.pdf]

Supporting Information

Synthesis of poly(olefin sulfone)s that release low-molecular-weight bases by light absorption and investigation of their photoinduced depolymerization

*Sumie Takemura, Atsushi Seki, Khoa Van Le, Yumiko Naka, and Takeo Sasaki**

Department of Chemistry, Faculty of Science, Tokyo University of Science, 1-3 Kagurazaka, Shinjuku-ku, Tokyo 162-8601, Japan

* sasaki@rs.tus.ac.jp

Contents

1. ^{1}H NMR spectra of monomers and polymers synthesized in this study (Figures S1–S5)
2. Gel permeation chromatography (GPC), differential scanning calorimetry (DSC), and thermogravimetric analysis of the polymers (Figures S6–S8)
3. Change in IR spectra of the polymers induced by photo-irradiation (Figures S9)
4. Change in IR spectra of the polymers induced by heating (Figures S10)
5. FT-IR spectra used for calculation of the decomposition rate of polymers (Figures S11–S13)
6. ^{1}H NMR spectra of polymers before and after UV irradiation (Figures S14–S15)
7. ^{1}H NMR spectra of copolymers (Figures S16–S21)
8. Differences in the change in film thickness when the film is irradiated with UV light from the air side and the glass substrate side (Figures S22 and S23)

1. ^{1}H NMR spectra of monomers and polymers synthesized in this study

The NMR spectra of the monomers were acquired using a JNM-ECA 300 MHz NMR spectrometer (JEOL, Japan).

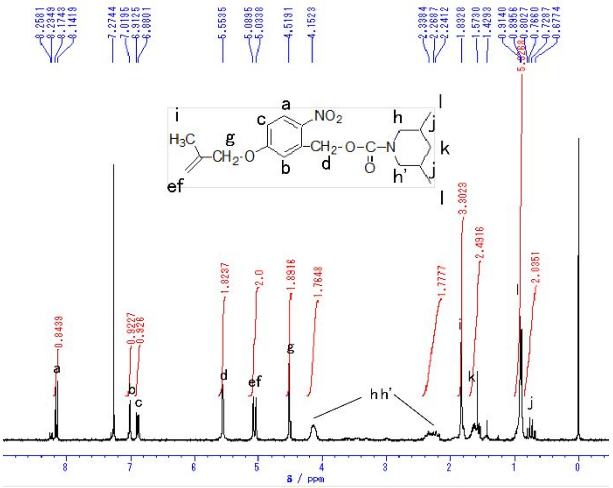

Figure S1. ^{1}H-NMR spectrum of the MNCDP monomer.

^{1}H NMR (300 MHz, CDCl$_3$, ppm) δ 7.97 (br, 1H, Ar-H), 6.99 (br, 1H, Ar-H), 6.77 (br, 1H, Ar-H), 5.41 (br, 2H, Ar-CH$_2$-O-), 4.64 (br, 2H, O-CH$_2$-C(CH3)=CH$_2$), 3.97 (br, 2H, N-CH$_2$), 2.15 (br, 2H, N-CH$_2$), 1.80-0.634 (br, 8H, CH$_2$, CH), 0.814 (br, 9H, C(CH$_3$))

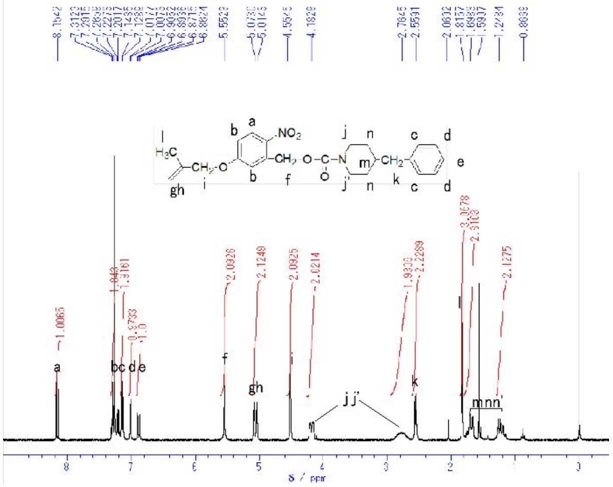

Figure S2. ^{1}H-NMR spectrum of the MNCBP monomer

^{1}H NMR (300 MHz, CDCl$_3$, ppm) δ 7.92(br, 1H, Ar-H), 7.21–6.77 (m,s 5H, Ar-H), 5.38 (br, 2H, Ar-CH$_2$-O-), 4.59 (br, 2H, O-CH$_2$-C(CH3)=CH$_2$), 4.00 (br, 2H, N-CH$_2$), 2.97 (br, 2H, N-CH$_2$), 2.64 (br, 2H, N-CH$_2$-Ar), 1.76 (br, 3H, CH$_2$, CH), 1.07 (br, 2H, N-CH$_2$)

^{1}H NMR spectra of the MNCDP, MNCBP, and MNCP polymers

The NMR spectra of the polymers were acquired using a JNM-ECA 300 MHz NMR spectrometer (JEOL, Japan).

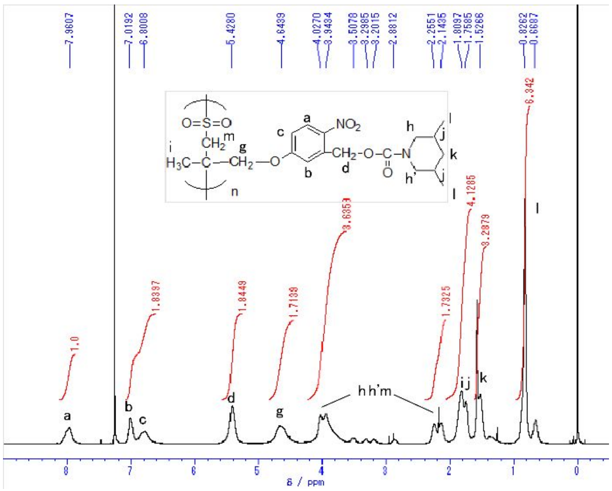

Figure S3. ^{1}H-NMR spectrum of the MNCDP polymer.

^{1}H NMR (300 MHz, $CDCl_3$, ppm) *δ* 7.97 (br, 1H, Ar-H), 6.99 (br, 1H, Ar-H), 6.77 (br, 1H, Ar-H), 5.41 (br, 2H, Ar-CH_2-O-), 4.64 (br, 2H, O-CH_2-C(CH_3)=CH_2), 3.97 (br, 2H, N-CH_2), 2.15 (br, 2H, N-CH_2), 1.80–0.634 (br, 8H, CH_2, CH), 0.814 (br, 9H, C(CH_3))

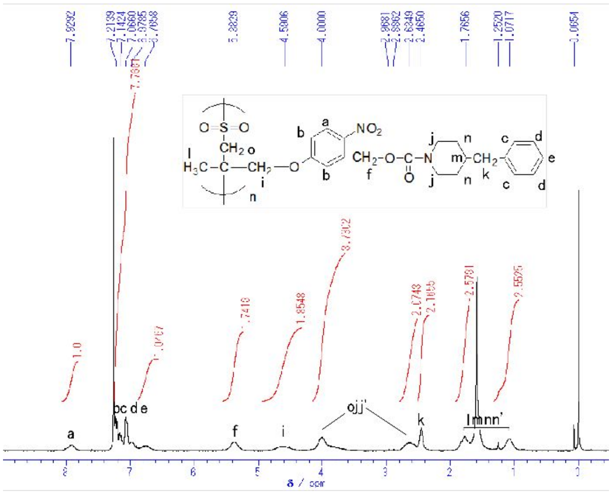

Figure S4. ^{1}H-NMR spectrum of MNCBP polymer.

^{1}H NMR (300 MHz, $CDCl_3$, ppm) *δ* 7.92(br, 1H, Ar-H) δ7.21-6.77 (m,s 5H, Ar-H) δ5.38(br, 2H, Ar-CH_2-O-) δ4.59(br, 2H, O-CH_2-C(CH3)=CH_2) δ4.00 (br, 2H, N-CH_2) δ2.97 (br, 2H, N-CH_2) δ2.64 (br, 2H, N-CH_2-Ar) δ1.76 (br, 3H, CH_2, CH) δ1.07 (br, 2H, N-CH_2)

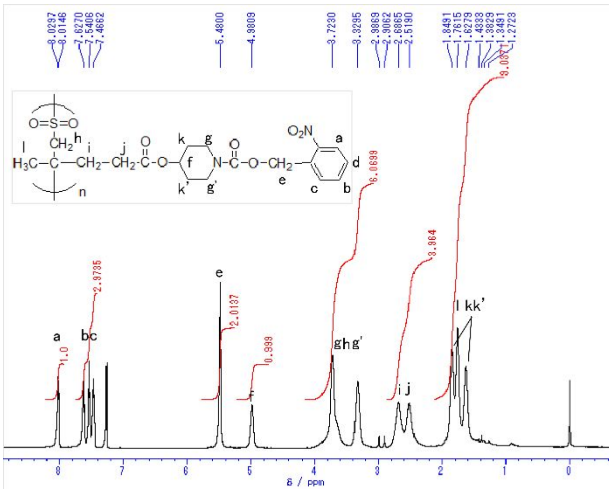

Figure S5. ^{1}H-NMR spectrum of MNCP polymer.

^{1}H NMR (500 MHz, CDCl$_3$, ppm) δ 8.02 (br, 1H, ArH), 7.47–7.63 (br, 3H, ArH), 5.48 (br, 2H, -CO-O-C$\underline{H}_3$-), 5.00 (br, 1H, -CO-O-C$\underline{H}$-), 1.66–1.87 (each br br, total 2H, -C$\underline{H}_2$-CH$_2$-N-), 1.75 (br, 3H, CH$_3$), 2.34 (br, 2H, -C$\underline{H}_2$-CH$_2$-C=O), 2.48 (br, 2H, -CH$_2$-C$\underline{H}_2$-C=O), 3.38–3.71 (each br br, total 4H, -N-C$\underline{H}_2$-, polymer chain)

2. Gel permeation chromatography (GPC), differential scanning calorimetry (DSC), and thermogravimetric analysis of the polymers

Gel permeation chromatography (GPC) of polymers

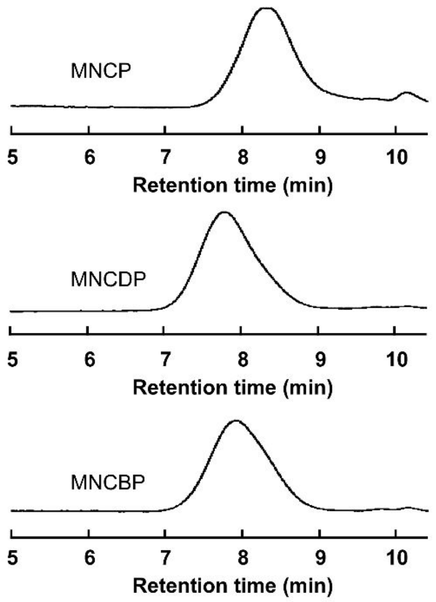

Figure S6. GPC curves of the MNCBP of polymers. Tetrahydrofuran (THF) was used as an eluent.

Differential scanning calorimetry (DSC) of the polymers

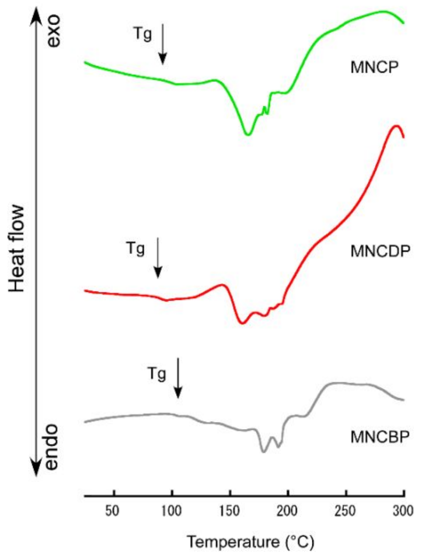

Figure S7. Differential scanning calorimetry (DSC) of the polymers. The heating rate was 10°C/min. The glass transition temperatures were determined from the first scan data of the DSC measurement because the poly(olefin sulfone)s thermally degraded during the measurement. Tg's were observed at around 80-105 °C. Decomposition of the side-chain photobase generator occurred at temperatures around 150 °C. Degradation of the polymer backbone and evaporation of the products occurred at higher temperatures.

Thermogravimetric analysis of the polymers

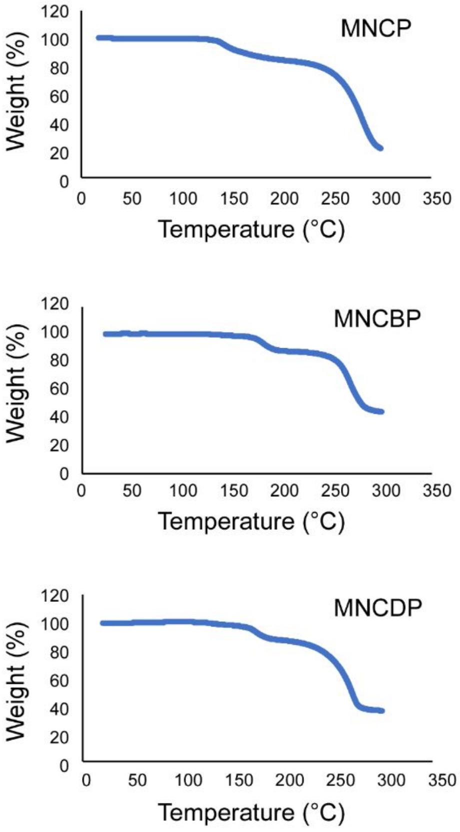

Figure S8. Thermogravimetric analysis of polymers. The decomposition of the polymer occurred after the thermal decomposition of the side-chain photobase generators, and the weight of the polymers began to decrease at 190-200 °C.

3. Change in IR absorption spectra of the polymers induced by photo-irradiation

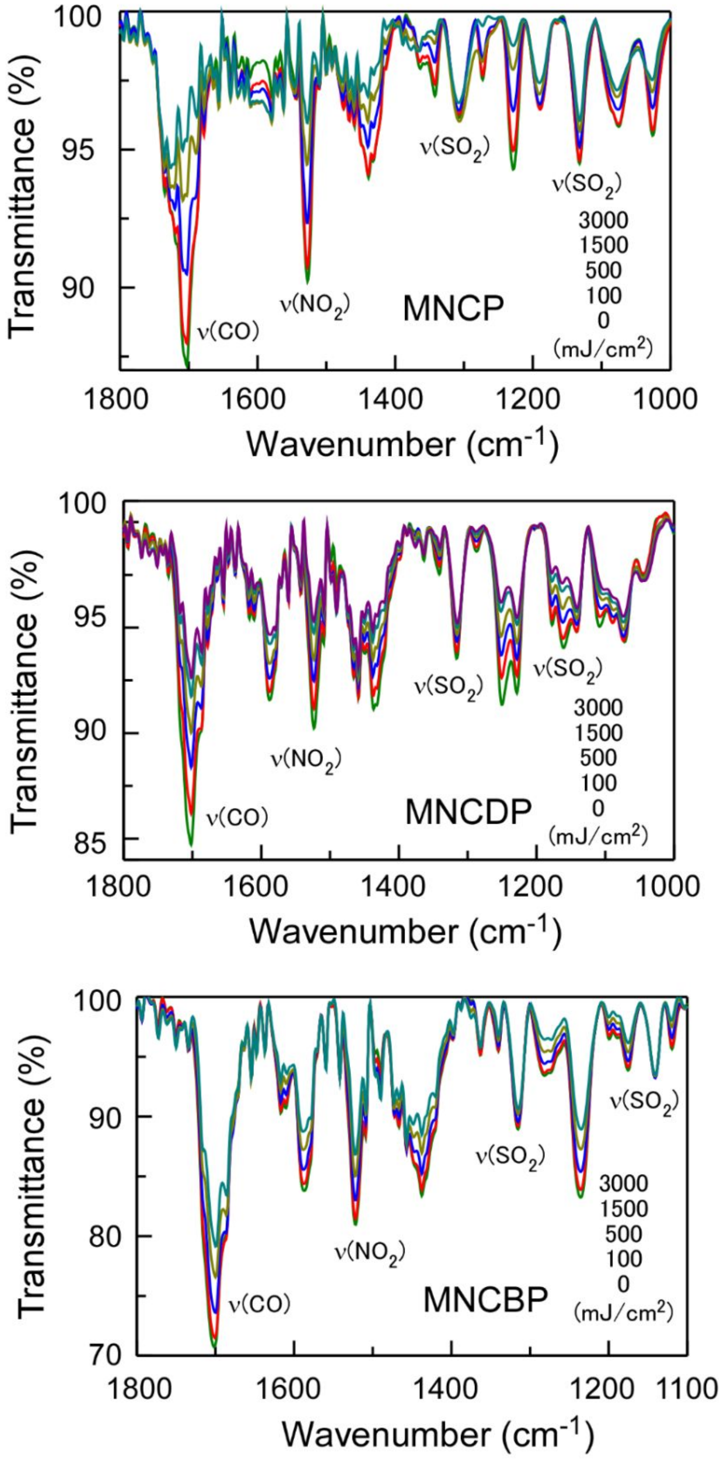

Figure S9. FT-IR absorption spectra of the MNCP, MNCBP, and MNCBP polymers after UV irradiation at room temperature.

4. Change in IR absorption spectra of the polymers induced by heating

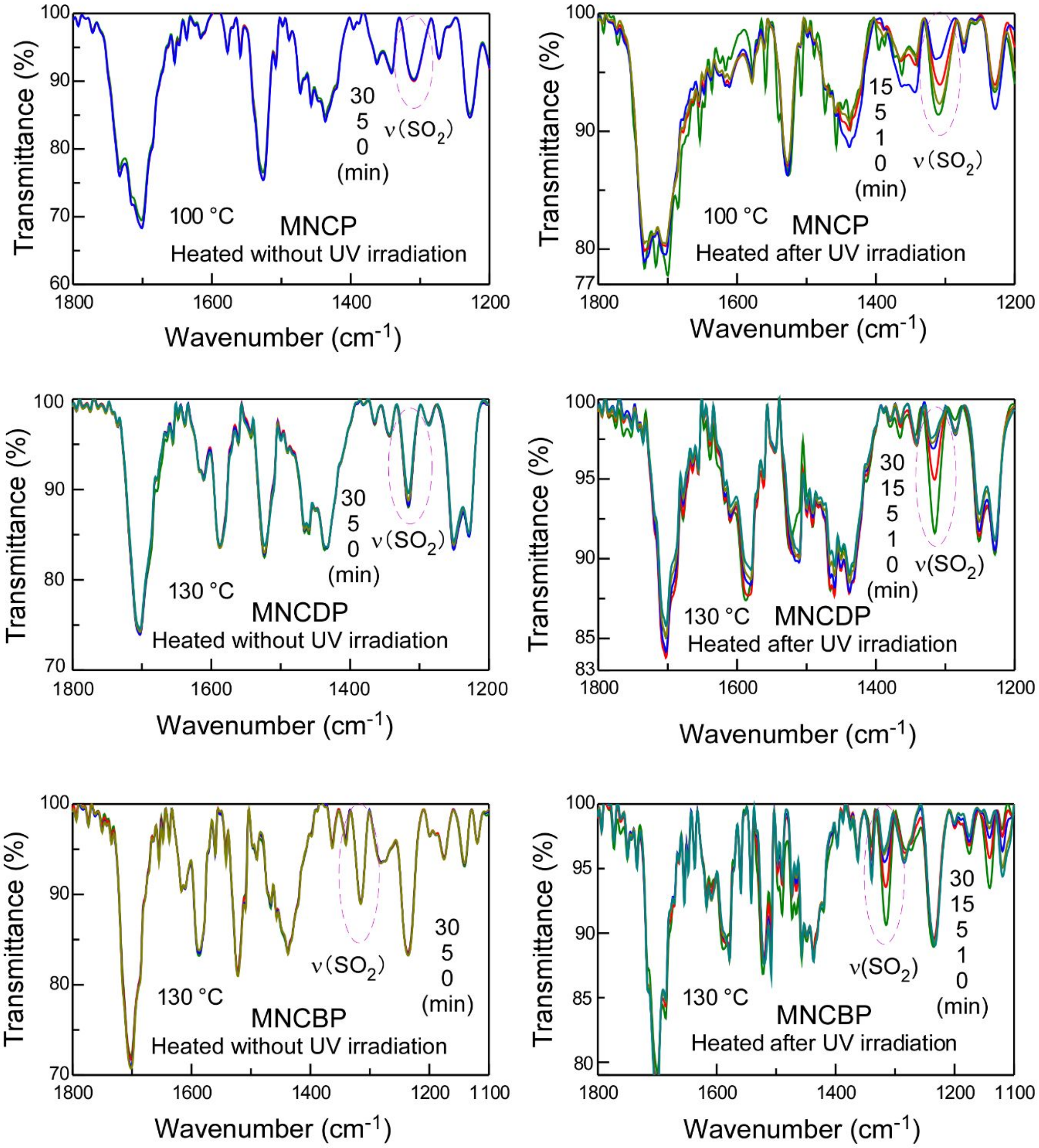

Figure S10 FT-IR spectra of the polymers heated without and after UV irradiation.

5. FT-IR spectra used for the calculation of the decomposition rate of the polymers

The decomposition rate was calculated from the IR absorption intensity of the sulfonyl moiety. The absorptions of the sulfonyl group of olefin monomers were subtracted from those of the polymers. The changes in this value were then studied over time and with exposure of the samples to UV radiation.

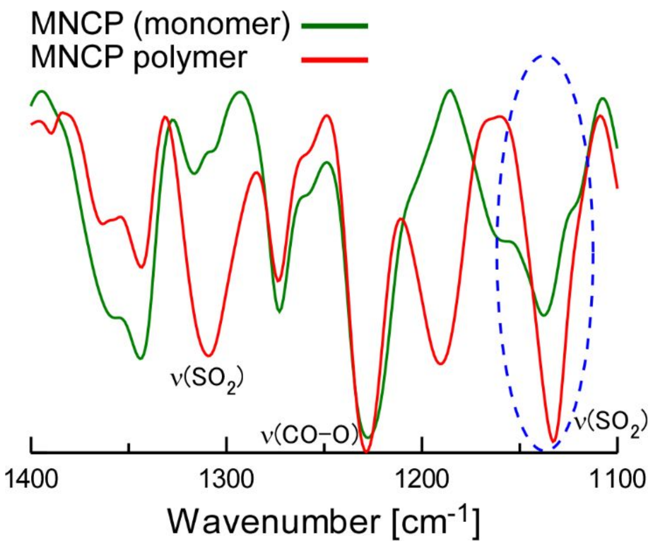

Figure S11. FT-IR absorption spectra of the MNCP monomer and the MNCP polymer.

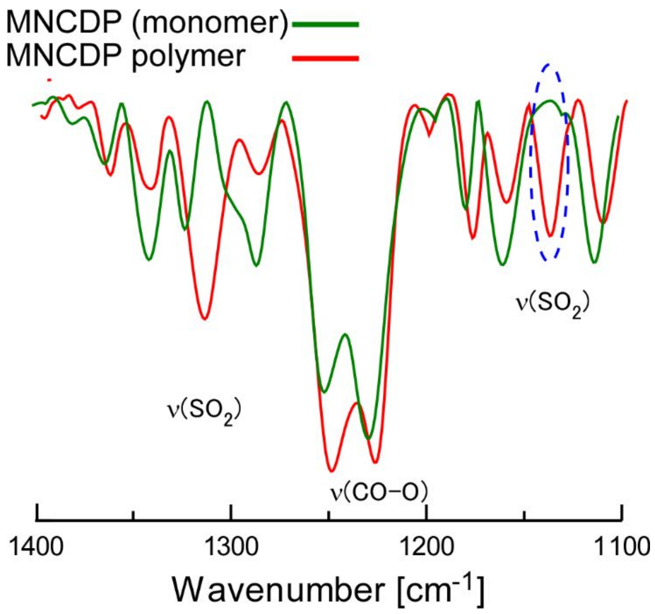

Figure S12. FT-IR absorption spectra of the MNCDP monomer and the MNCDP polymer.

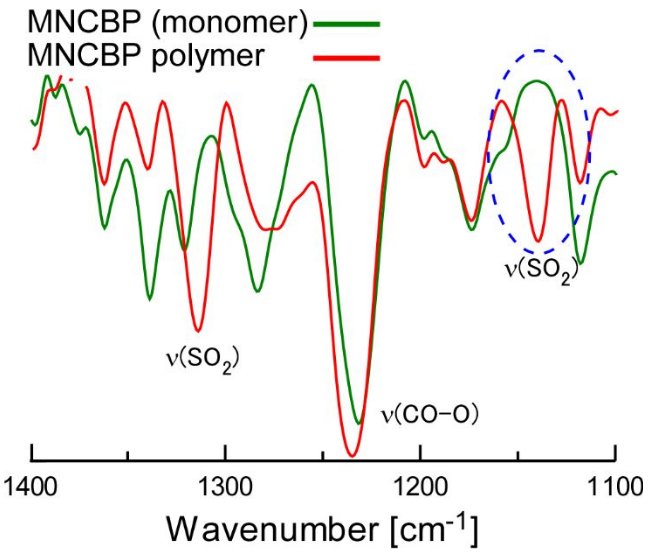

Figure S13. FT-IR absorption spectra of the MNCBP monomer and the MNCBP polymer.

6. Confirmation of the photoinduced depolymerization of polymers

When the polymer films were irradiated with UV light and then heated, their ^{1}HNMR spectra changed substantially. The signals that appeared after light irradiation matched those in the NMR spectrum of the monomer.

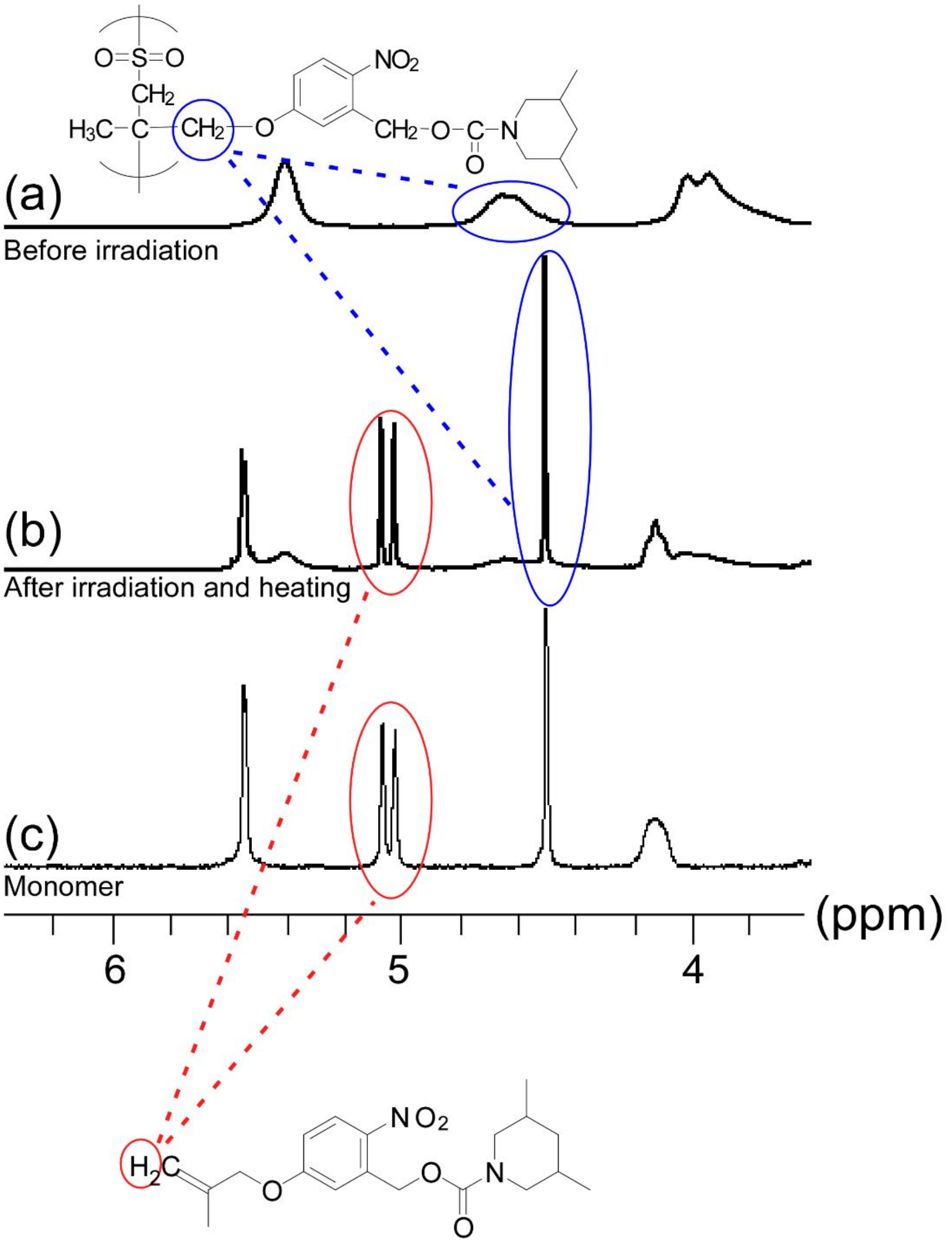

Figure S14 ^{1}H-NMR spectra of chloroform-*d* solutions of (a) the MNCDP polymer before UV irradiation, (b) the MNCDP polymer after UV irradiation at 3000 mJ/cm^{2} followed by heating at 130 °C for 30 min, and (c) the MNCDP monomer.

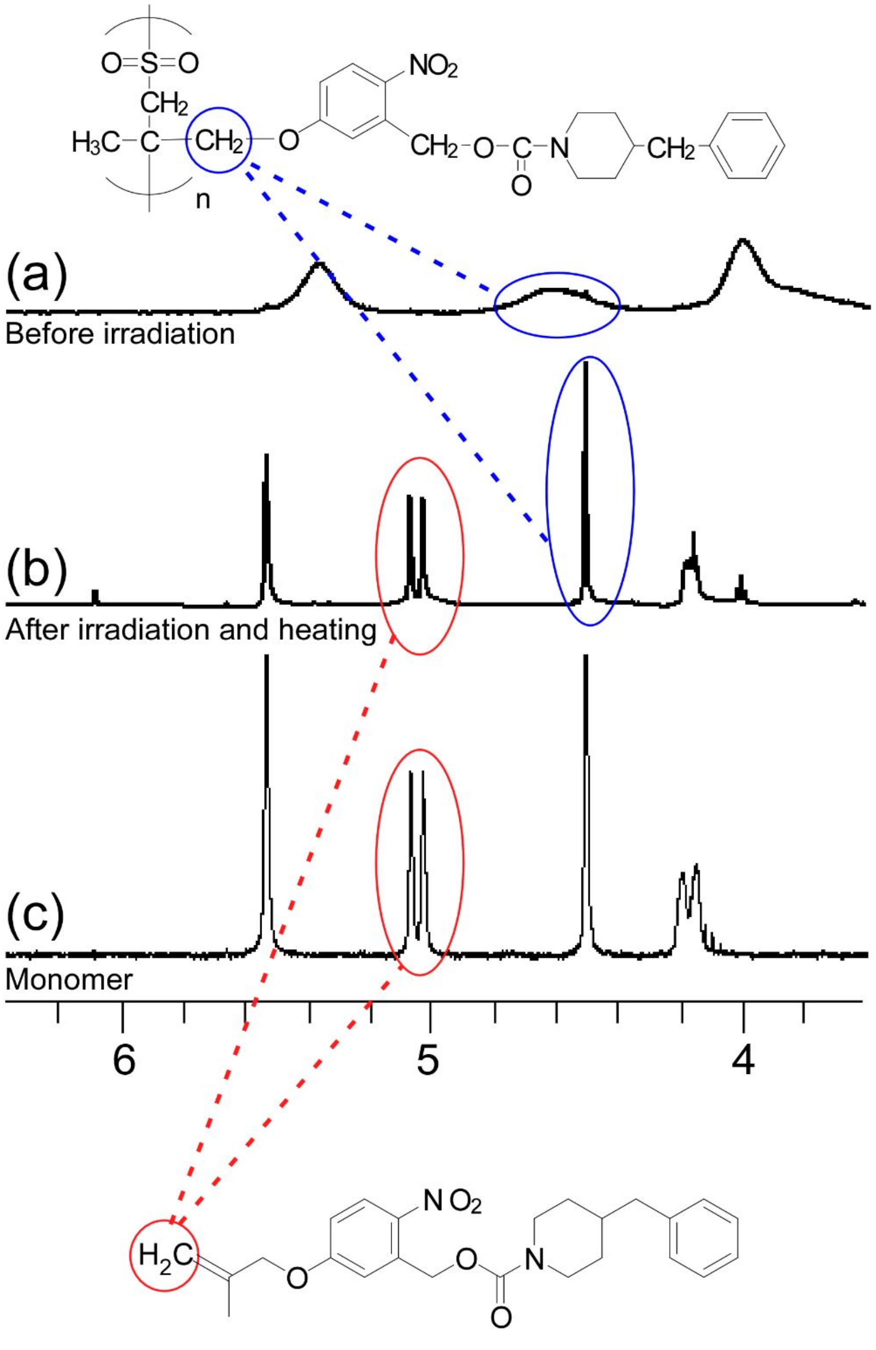

Figure S15. ^{1}H-NMR spectra of chloroform-*d* solutions of (a) the MNCBP polymer before UV irradiation, (b) the MNCBP polymer after UV irradiation at 3000 mJ/cm^2 followed by heating at 130 °C for 30 min, and (c) the MNCBP monomer.

7. ¹H NMR spectra of copolymers Co-MNCBP1, Co-MNCBP2, and Co-MNCBP3

The copolymerization ratios were calculated using the integral of the signals shown in the figures.

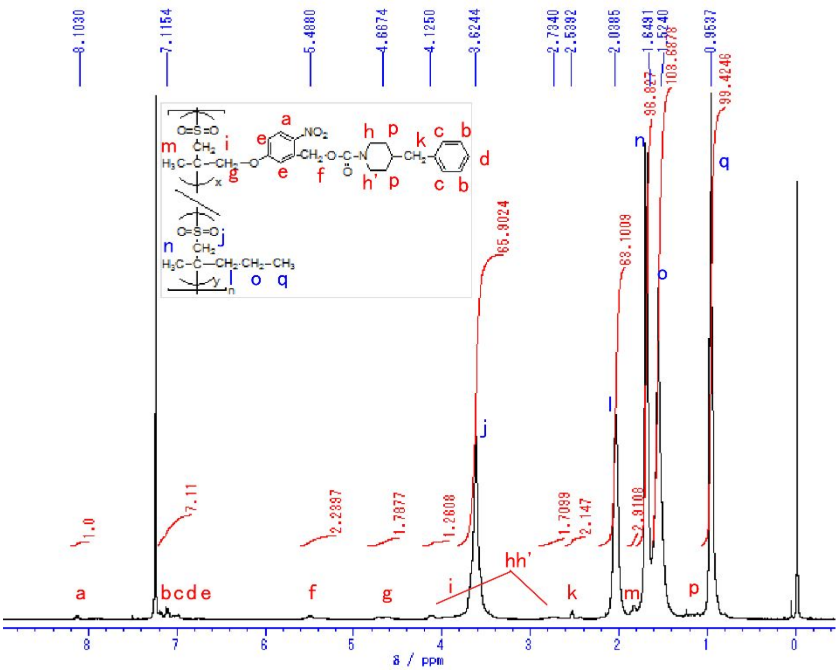

Figure S16. ¹H-NMR spectrum of Co-MNCBP1.

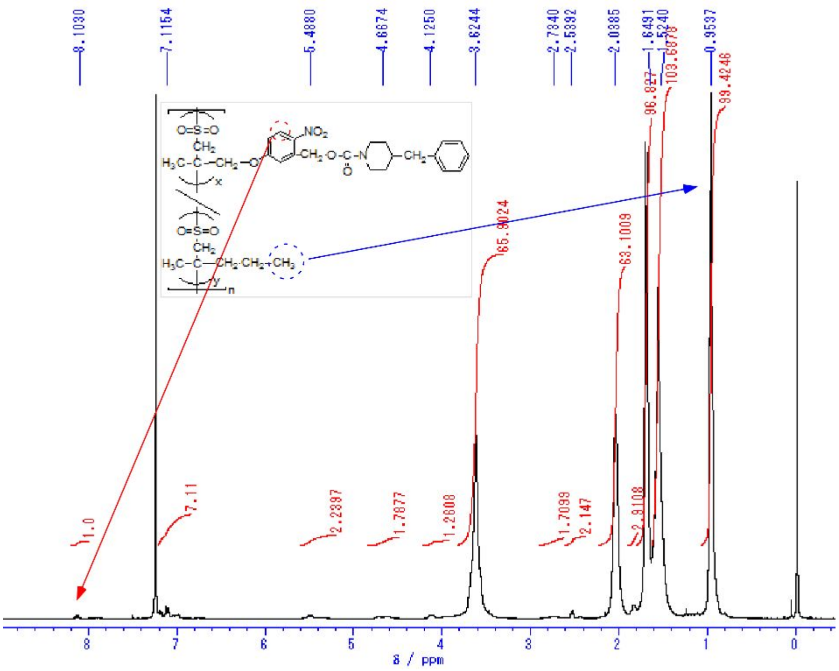

Figure S17. ¹H-NMR spectrum of Co-MNCBP1. The copolymerization ratio was calculated from the signals indicated by the arrows.

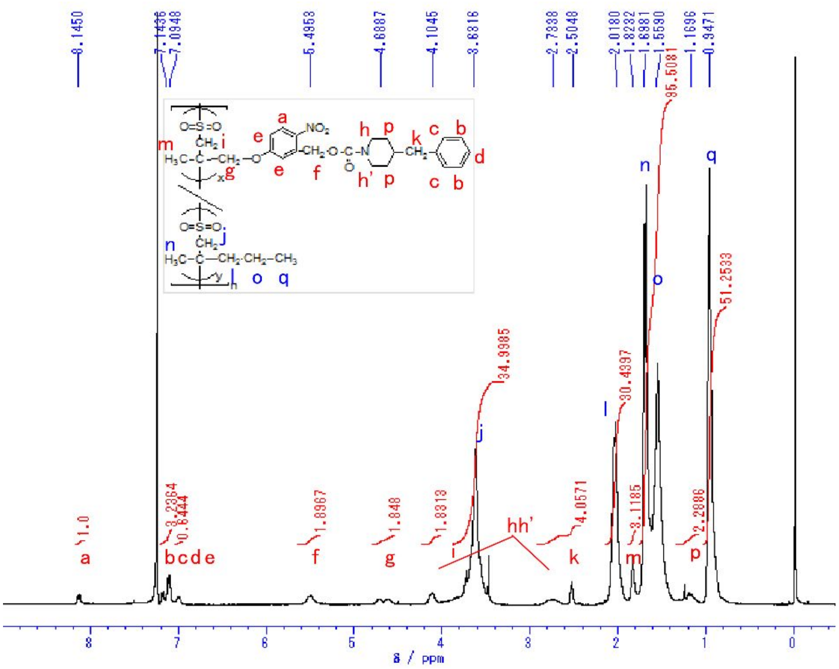

Figure S18. ^{1}H-NMR spectrum of Co-MNCBP2.

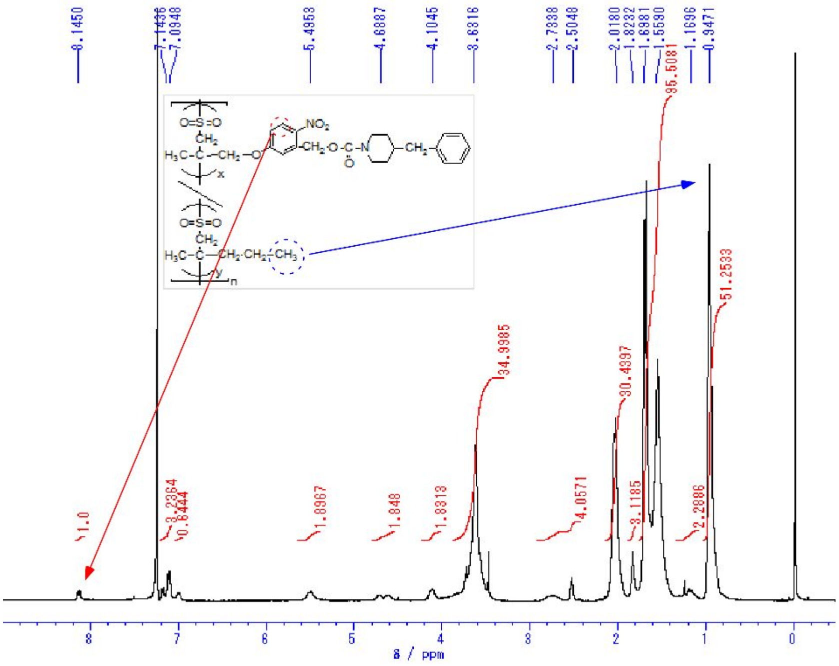

Figure S19. ^{1}H-NMR spectrum of Co-MNCBP2. The copolymerization ratio was calculated from the signals indicated by the arrows.

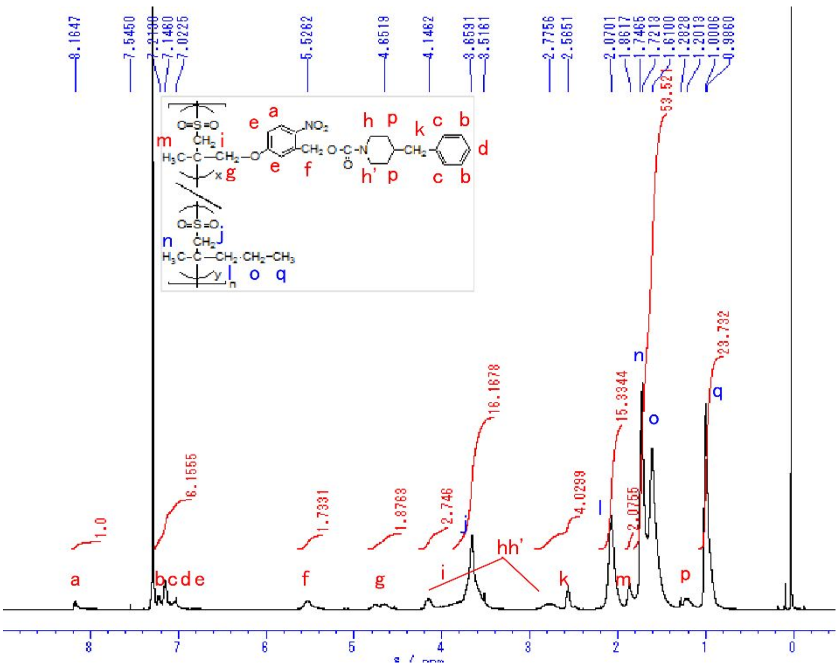

Figure S20. ¹H-NMR spectrum of Co-MNCBP3.

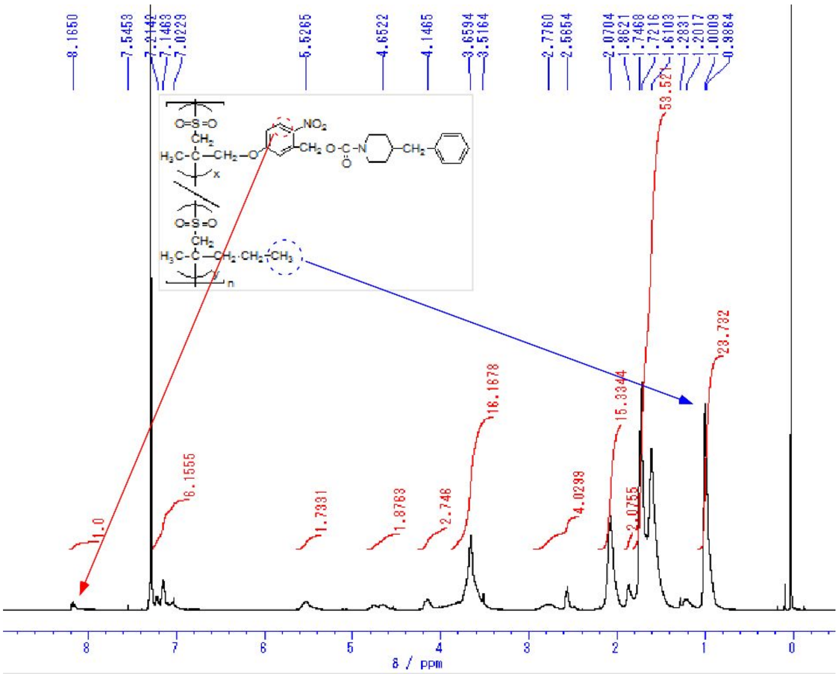

Figure S21. ¹H-NMR spectrum of Co-MNCBP3. The copolymerization ratio was calculated from the signals indicated by the arrows.

8. Differences in film thickness change under irradiation by UV light from the air side and from the glass-substrate side of the film

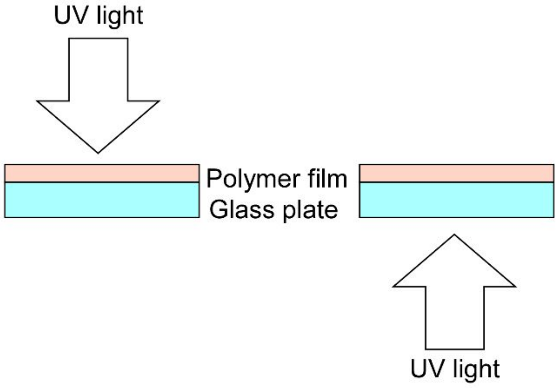

Figure S22. UV light irradiation from the air side and the glass-plate side.

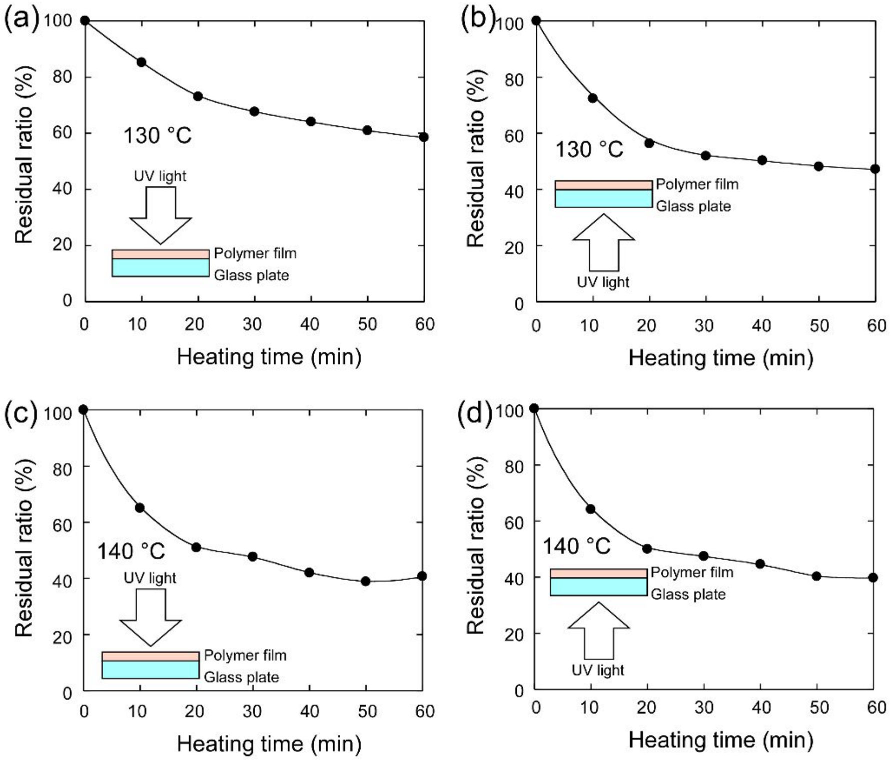

Figure S23. Residual ratios for the copolymer film, as measured by AFM, plotted as a function of heating time. (a) Film irradiated with UV light at 5000 mJ/cm^2 from the air side and heated at 130 °C; (b) film irradiated with UV light from the glass-plate side and heated at 130 °C; (c) film irradiated with UV light from the air side and heated at 140 °C; (d) film irradiated with UV light from the glass-plate side and heated at 140 °C. The polymer used was a copolymer of MNCBP, 2-methylpentene, and SO_2. The copolymerization ratio of MNCBP:2-methylpentene was 1:12, the number-average molecular weight (M_n) was 93,000, and the degradation temperature (T_d, 10% loss) was 157 °C.
